# The Method of Everything vs. Experimenter Bias of Loophole-Free Bell Experiments

**DOI:** 10.3389/frma.2024.1404371

**Published:** 2024-07-11

**Authors:** Manuel S. Morales

**Affiliations:** Science, Math, Technology Division, Rowan College at Burlington County, Mount Laurel, NJ, United States

**Keywords:** Method of Everything, double-slit experiment, Bell inequalities, EPR Paradox, non-local hidden variables, superdeterminism, experimenter bias, artificial intelligence

## Abstract

Experimenter bias compromises the integrity and advancement of science, especially when awarded as such. For example, the 2022 Nobel Prize in Physics awarded for the loophole-free experiments that tested physicist John S. Bell's inequality theorem. These experiments employed the logic of conducting local experiments to obtain local evidence that contradicted local realistic theories of nature, thereby validating quantum mechanics as a fundamental non-local theory. However, there was one loophole that was wittingly not tested by the Nobel laureates. The notable exception was Bell's “super-deterministic” loophole, which was validated (2000) (2001) (2002) (2003) (2004) (2005) (2006) (2007) (2008) (2009) (2010) (2011) (2012) non-locally, thus compromising the subsequent Nobel Prize. More importantly, the discovery of two mutually exclusive and jointly exhaustive non-local hidden variables revealed why local scientific methods obtain false-positive and false-negative results. With knowledge of this fundamental omission, the inclusion of the non-local hidden variables in the local methods used in science can then advance it to be a complete study of nature.

## 1 Introduction

Ever since the heated discussions between Albert Einstein and Niels Bohr at the Solvay Conference in 1927 (Fine and Ryckman, 2020), the question of whether or not quantum mechanics is a fundamental theory has been highly debated. To further address this dispute, Albert Einstein and physicists Boris Podolsky and Nathan Rosen published a study in 1935, “Can Quantum Mechanical Description of Physical Reality Be Considered Complete?” aka the Einstein-Podolsky-Rosen (EPR) Paradox (Einstein et al., 1935). In 1964, the physicist John S Bell contested Albert Einstein's suggestion of local hidden variables (Belousek, 1996) using his theorem in the article titled “On The Einstein Podolsky Rosen Paradox” (Bell, 1964). Over the following decades, physicists Alain Aspect, John F. Clauser, and Aton Zeilinger tested the loopholes of Bell's theorem and subsequently were awarded the Noble Prize in Physics in 2022 for their local experiments (local input–cause–local output), thus validating the assumption that quantum mechanics is a fundamental non-local theory (The Nobel Prize in Physics 2022, 2022). Although the Nobel laureates closed several loopholes of Bell's theorem with their local experiments, there was one notable exception—Bell's super-deterministic loophole (Brans, 1988)—which was wittingly not closed.

In layman's terms, the near-century-old argument has been about cause and effect and whether the said function is local or non-local. It is also about the validity of the methods applied and which logic code correlates with the data/empirical evidence obtained. The ramification of this debate not only affects science but also affects our knowledge of how the physical universe and our existence came to be. This brings us to the topic at hand: experimenter bias vs. the Method of Everything.

As a product of nature, mankind is bound by its laws. Therefore, mankind cannot change the laws that nature has predetermined before our existence. This includes the predetermined laws that enable mankind to study nature. Herein lies the folly of the logic that local *effects* of existence *cause* the local existence of its *effects*, which I define as ***E-C*** logic, i.e., local ***E****ffects*–***C****ause*–local *effects*. As such, effects are causal and the cause is effectual, thus serving as an interaction (second-order function) between effects. This logic is fundamentally incomplete, for it assumes states of existence that can be observed or measured are self-causal, for example, states of energy being conserved and thus cannot be created or destroyed. In essence, E-C logic is self-contradictory such that it would necessitate the reader's existence caused by its own existence, and therefore, human beings are conserved states of existence that cannot be born, much less have parents. The paradox of E-C logic is that if this is how nature works, then human beings are anomalies of nature. If not, then the logic applied in systematic methods to study nature and to test physical theories is invalid or at best incomplete. We cannot have it both ways.

In the non-local no-go Tempt Destiny Experiment (2000–2012) (Morales, 2011a), the bias of using local input to cause local output, as previously exemplified, has been shown to hide the non-local mechanisms of nature (Tables 1–3) necessary to conduct all local experiments. This discovery means that local hidden variables, which Albert Einstein had predicted nearly a century ago, are in fact non-local. The evidence obtained supports Einstein's suggestion that all variables should be accounted for. The findings reveal that quantum mechanics is a local effect of non-local hidden variables. This means that quantum mechanics is indeed an incomplete theory, as Einstein had argued. Because current empirical methods based on E-C logic are incomplete, as exhibited in Table 2, we now know why false-positive and false-negative empirical evidence is obtained.

**Table 1:** Tempt Destiny Experiment—Method of Everything vs. incomplete methods.

**Causal**	**Non-local Input–Cause–Local Output Cause-Effect (C-E logic)**	**Self-Causal**	**Local Input–Cause–Local Output Effect-cause (E-C logic)**
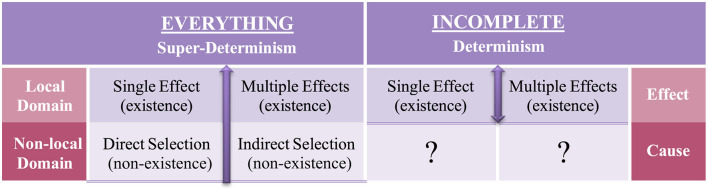

**Table 2:** Unambiguous empirical evidence of predetermined choice.

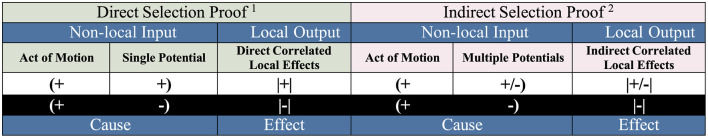

The Method of Everything, as applied in the Tempt Destiny (TD) experiment, tested both mutually exclusive and jointly exhaustive non-local selection functions of motion (+) and the falsification (–) thereof. The direct cause–effect correlation makes the evidence unambiguous and its falsification absolute. As confirmed, direct selection and indirect selection are dichotomous mechanisms of motion. Therefore, it is necessary that both contradictory non-local components, motion, and their mutually exclusive and jointly exhaustive potential functions must be simultaneous; otherwise, a selection event cannot come to exist as noted |-|.

As observed, no selection event (+, –) occurred in 11 out of 12 direct selection tests (Bell, 2004), confirming that selection functions are predetermined non-local mechanisms of motion such that they can only come to exist not preexist non-locally, or be locally existent. This means that they are intrinsically fundamental and thus causal by design, which explains why it is impossible to conduct any local experiment without said functions.

^1^(+) When an act of motion is paired with a single potential (+, +), the effect is a direct selection = |+|. This event (+, +) = |+| occurred once out of 12 attempts.

(–) When an act of motion is not paired with a single potential (+, -), the effect is no direct selection = |-|. This event (+, -) = |-| occurred in 11 out of 12 attempts.

^2^(+) When an act of motion is paired with more-than-one potential (+, +/–), the effect is an indirect selection = |+/-|. This event (+, +/-) = |+/-| occurred in three out of three attempts.

(–) When an act of motion is not paired with the more-than-one potential (+, -), the effect is no indirect selection = |-|. This event (+, -) = |-| almost occurred in one of the three attempts.

As previously stated, the logic code currently used to study nature has been empirically proven, without exception, to be inverted (backward), thus a violation of how the laws of nature work. For example, instead of using E-C logic, I used what I define as ***C-E*** logic, i.e., non-local ***C****ause* preceding local ***E****ffect*. As exhibited in Table 2, unambiguous empirical evidence confirmed that the non-local domain of motion precedes local effects of existence; existence does not precede effects of motion. Section 7.2 shows how the non-local acts of motion were paired with direct and indirect potential functions, without which the effect of a local experiment could not occur. The experiment revealed that two mutually exclusive and jointly exhaustive variables of motion are predetermined to only come-to-exist which means that they cannot preexist non-locally or be locally existent (Morales, 2016). This is what makes them intrinsically hidden, non-local, and predetermined prior to the effects of existence. Section 6 shows how current methods and theories based on E-C logic necessarily fail to account for said variables as first-order functions, thus hiding them as second-order effects.

Since the findings are universal and unambiguous, all of humanity can test the findings for themselves via the following Final Selection Experiment (FSE), first published in 2013, to confirm the external validity of the Tempt Destiny Experiment (Morales, 2013). It should be noted that although testable (FSE), the method applied is absolute and all-inclusive and thus not subject to statistical inference, conjecture, or theory. As such, the focus of this manuscript is on method validity dictated by initial conditions, i.e., what comes first. I defer details of the theories mentioned to cited references for those who wish to explore further.

If the reader subscribes to the principle that only empirical evidence, rather than philosophical opinions, beliefs, mathematics, or theories, can negate and thus invalidate empirical evidence, then the subsequent findings will be of interest. Those who do not support the empirical principle of what makes science a valid study of nature need not read further.

## 2 The Method of Everything

*How do we know what we think we know?* Was the central question I needed to ask myself when trying to make sense of the non-local no-go Tempt Destiny Experiment (non-local input–cause–local output) that I conducted from 2000 to 2012 to test whether destiny, also known as superdeterminism, was valid or not (Morales, 2011b,c). Instead of using local existence as a causal function (local input–cause–local output), as currently practiced in science, I used the mutually exclusive and jointly exhaustive non-local functions of motion, i.e., direct selection and indirect selection—otherwise known as choice, as first-order No-Go functions to conduct the local experiment (Table 1). Note that if the universe is indeed “super-deterministic” as physicist John S Bell coined (Davies and Brown, 1986), then the mechanics of how a choice can be made must also be predetermined by nature as a first-order function, not as a second-order cognitive function determined by the experimenter. After all, the experimenter is a product of nature, not the cause of it.

By applying C-E logic as the method to conduct the Tempt Destiny Experiment, the protocol included both mutually exclusive domains of nature, i.e., existence and non-existence, in their proper order. Since the experiment obtained unambiguous empirical evidence via *direct correlation* of the cause preceding effect, the findings confirmed the primary domain and the secondary domain. This means that local empirical evidence is non-locally predetermined, not locally determined. This is how nature obtains something (existence) from nothing (non-existence). As such, all of humanity can confirm the findings for themselves via the FSE.

Following the conclusion of the Tempt Destiny Experiment in January 2012, CERN announced their preliminary discovery of the Higgs boson in July 2012 (CERN, 2012). I then contacted CERN physicists to see how or if their local Large Hadron Collider (LHC) experiment accounted for the two predetermined non-local mechanisms of selection that are necessary to conduct all local experiments (Aad et al., 2012). As the correspondence confirmed, this was not the case. Thus, a serious omission error (experimenter bias) was made in conducting their experiment, subsequently compromising the validity of their data (Table 3—Hidden Variables^†^).

**Table 3:** Predetermined outcomes—false-positive and false-negative results.

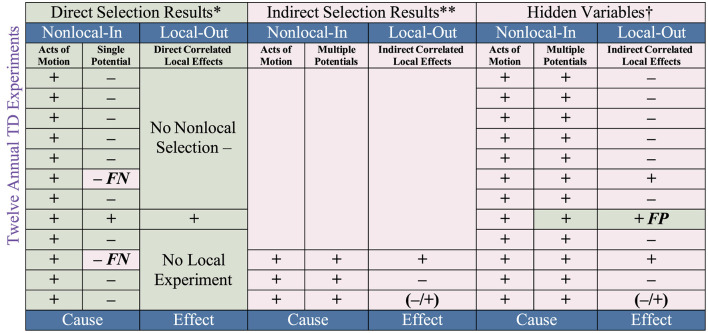

^*^Evidence of an act of motion paired with a single potential–direct selection–certain outcome. ^**^Evidence of an act of motion paired with a more-than-one potential–indirect selection–uncertain outcome. **(–/+)** The SB XLVI painting was canceled; thus, no potential completion (**–**) by the team that eventually won (**+**). In the last 3 years of the TD experiment, if the primary potential option of a direct selection did not occur, then an indirect selection became the secondary potential option (see Section 7.2 for details).

^†^Note, if an indirect selection was used exclusively to conduct the TD experiment, 12 out of 12 attempts would have occurred, including the direct selection event as noted **+**FP. This means that indirect selection experiments, such as LHC experiments, inherently hide direct selection effects, thereby generating false-positive **+**FP empirical evidence (Morales, 2011b). In addition, if the TD experiment only used direct selection for the entire 12-year span of the experiment, two positive false-negative, FN, results would have been hidden.

## 3 Ethics of scientific discovery

When an empirical discovery supersedes previous knowledge, it is the responsibility of the practitioners of the art to test the breakthrough and, if valid, accept such knowledge for the advancement of science. Failure to do so compromises the integrity of science by preventing its advancement and, in this case, compromises the legitimacy of science by wittingly ignoring the discovery of non-local hidden variables.

In accordance with those ethics, I subsequently contacted physicists at CERN and the Nobel Committee at the time of my discovery and submitted my findings for peer review (Morales, 2012). After publication, I contacted both CERN and the Nobel Committee about the published findings. Nonetheless, later that year (2013), the Nobel Prize in Physics was awarded for the local LHC indirect selection experiment (*more-than-one* proton into *more-than-one* proton) of the Higgs boson discovery based on false-positive results (see Table 3) that exceeded, via 5σ, (Lyons, 2013) the absolute uncertain function threshold of more than one (99%). In other words, the absolute certainty threshold of one (1%), i.e., direct selection, cannot be a percentage of one or a percentage of more than one, e.g., 99.999%–5σ, since certainty (1%) and uncertainty (99%) are mutually exclusive and jointly exhaustive (100%). By applying Bell's inequality threshold of ≤ 2 to the experimental results of the Tempt Destiny Experiment, the 1% certainty threshold was not violated as per the Method of Everything ≤ 1 inequality in Section 7.3. Note that this award marked the first official recognition of experimenter bias as an acceptable practice to conduct science, thus establishing the precedence for continued awards of experimenter bias to follow.

## 4 Experimenter Bias of Loophole-Free Bell Experiments

*What good are theories if they are not fully tested?* I sought to address this question and the following question at the QUEST 2023 Conference in Paris, France, on 29 June 2023, where I was scheduled to speak in the same session with Nobel laureate Alain Aspect. Professor Aspect's lecture titled “Single Photons, Entangled Photons: From Quantum Foundations to Quantum Technologies” (Aspect, 2023) relates to his 2022 Nobel Prize in Physics. My lecture, “Experimenter Bias of Loophole-Free Bell Experiments”, as this article summarizes, pertains to the local (local input–cause–local output) indirect selection (beam splitter) experiments by Nobel laureates, including Alain Aspect, Anton Zeilinger, and John Clauser (Advanced Information, 2023), all of whom failed to address and thus close the non-local super-deterministic loophole, which is also known as, the “free-will” or “freedom of choice” loophole of physicist John S Bell's theorem (Bell, 2004). Note that Bell's theorem served as the basis for the awarded experiments (Bert and Evans, 2022).

“*Here is the loophole: Maybe, there is in the backwardv cones of ourselves or of our lives, some common events which decide how we are going to set the polarizers, our choice is not really free … I don't want to be a physicist in that world.”* – Alain Aspect (Phillips and Dalibard, 2023).

“*But, we maintain, skepticism of this sort will essentially dismiss all results of scientific experimentation. Unless we proceed under the assumption that hidden conspiracies of this sort do not occur, we have abandoned in advance the whole enterprise of discovering the laws of nature by experimentation.”* – J. F. Clauser (Shim, 1976).

“*The theory that the entire experiment, including choices and outcomes, is pre-determined by initial conditions is known as superdeterminism. Superdeterminism cannot be tested.” –* Anton Zeilinger et al. [The BIG Bell Test Collaboration]. (Zeilinger et al., 2018).

Nonetheless, the Tempt Destiny Experiment successfully obtained unambiguous empirical evidence from 2000 to 2012 that “choices” (Table 2) and their “outcomes” (Table 3) are indeed predetermined by (non-local) initial conditions (motion) as Anton Zeilinger specified yet objected to. This means that the 2022 Nobel Prize in Physics for the Bell-type experiments has been empirically invalidated since 2000.

The Bell-type experiments unwittingly used indirect selection, i.e., an act of motion (local photon beam) paired with more-than-one potential (local beam splitters), to conduct their local experiments. The unambiguous empirical evidence obtained from the Tempt Destiny Experiment revealed that all local empirical evidence is predetermined by two mutually exclusive and jointly exhaustive non-local selection functions of motion that are necessary to conduct all local experiments (Table 2). As such, obtaining a violation of Bell's theorem and or CHSH inequalities (Clauser et al., 1969) would correspond with obtaining false-positive indirect selection results (see datasheet). If the experimenters used direct selection to conduct their investigations, treating the local input of the photon beam as a single potential, not as a more-than-one potential via beam splitters, the predetermined results would be a single output, not a more-than-one output. In other words, a super-deterministic universe predetermined that a single local input = single local output, and multiple local input = multiple local output. By ignoring the non-local functions of motion that are necessary to conduct all local experiments, the Bell-type experiments are incomplete because of the experimenter's bias of assuming that the effects of existence (photon beam) are causal instead of the acts of motion as causal. This is what happens when the logic experimenters use (Existence precedes Cause—E-C logic) hides cause as an effect while chasing after empirical evidence to suit their predictions, thereby ignoring which mutually exclusive and jointly exhaustive selection mechanism was used to obtain their results.

As the evidence from the Tempt Destiny Experiment revealed, it is paramount not to ignore the significance of a super-deterministic universe to avoid obtaining erroneous empirical evidence. In such a universe, everything, existence, and non-existence are predetermined by nature, including the experimenter's choice to conduct one experiment as opposed to another (Table 3). A postulate that most, if not all, scientists find repugnant, as noted by Nobel laureate Anton Zeilinger in his lecture at the 2023 American Physical Society March Meeting, where he stated, “If our decisions were predetermined then why do we do experiments?” (Zeilinger, 2023). Excuses aside, if we are to investigate nature to understand it, then the bias of not testing whether the universe is super-deterministic or not relegates science as dogma (Nissen et al., 2016), subject to experimenter bias as awarded, not science conforming to the laws of nature.

## 5 All inclusive domains of the universe and their proper order

*What good are tests if they are not complete?* Nature, not the experimenter, dictates *what* can be tested but, more importantly, predetermines the *necessary order and mechanics of how* such tests can be executed. Therefore, for a test of nature to be complete, both domains of the universe, existence and non-existence (Table 1), need to be accounted for and necessarily in their proper order so that one domain does not obscure the function of the other, thereby creating a situation of hidden variables, e.g., direct selection hiding indirect selection and vice versa as shown in Table 3. Regarding the above testing parameters put forth, I refer to the domain of existence, i.e., “*what”*, effects that can be locally observed or measured in time and space, and the domain of non-existence, i.e., “*how”*, the non-local functions of motion (direct selection and indirect selection), as causal mechanisms. As previously mentioned, direct selection and indirect selection can only *come to exist* not preexist non-locally, or be locally existent (Table 2); as such, they are non-local functions of the first order. Accordingly, the *findings distinguished non-existence as a causal domain and existence as a domain of its effects*. Although both domains are mutually exclusive, they are all inclusive of the phenomenon we call the universe, i.e., everything. As empirically confirmed without ambiguity via the Method of Everything (Tables 1–3), this means that a *Theory of Everything* (Weinberg, 1994) is no longer a theory.

Absolute internal validity has confirmed that *the non-locality of motion causes the locality of its effects* (Cause precedes Existence—C-E logic). However, for this claim to be complete, it must also be tested for absolute external validity. To test for said validity, it is important to consider which logic code provides a direct cause–effect correlation without ambiguity.

## 6 The logic code of experimenter bias

**Figure 1:**
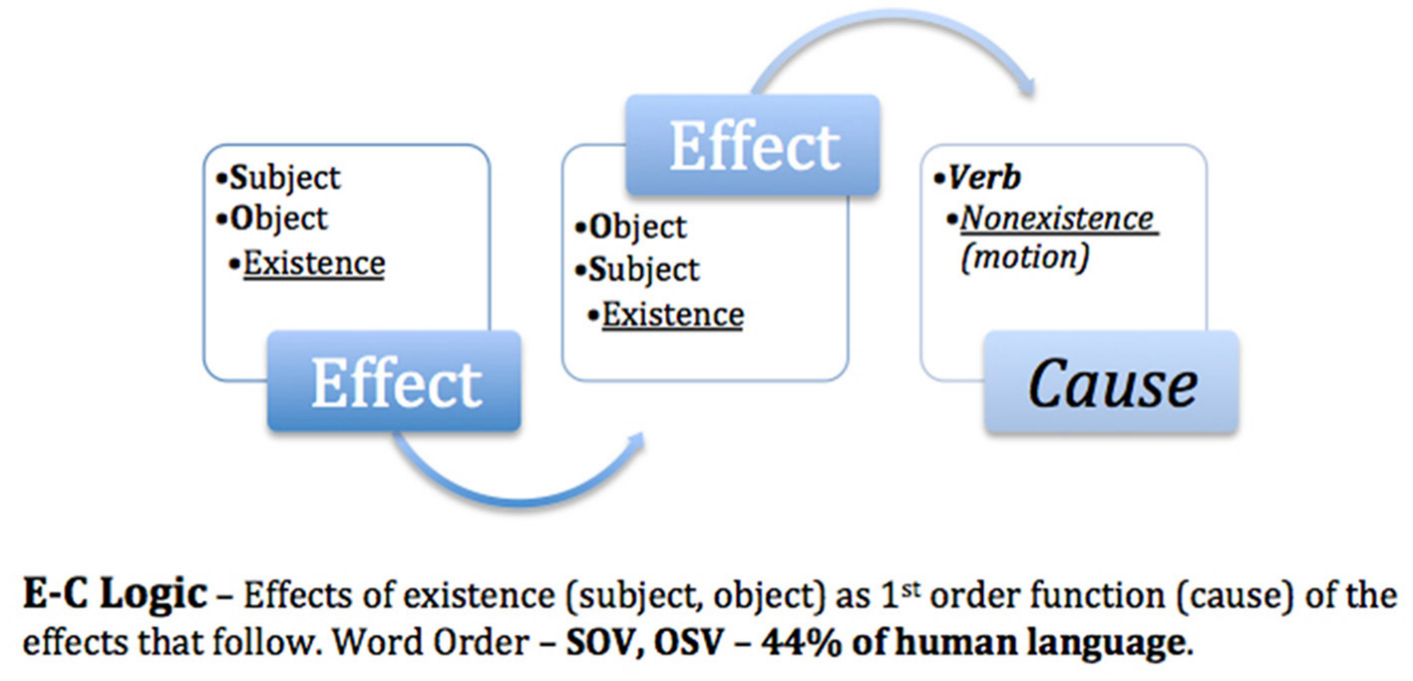
Effects precedes cause.

**Figure 2:**
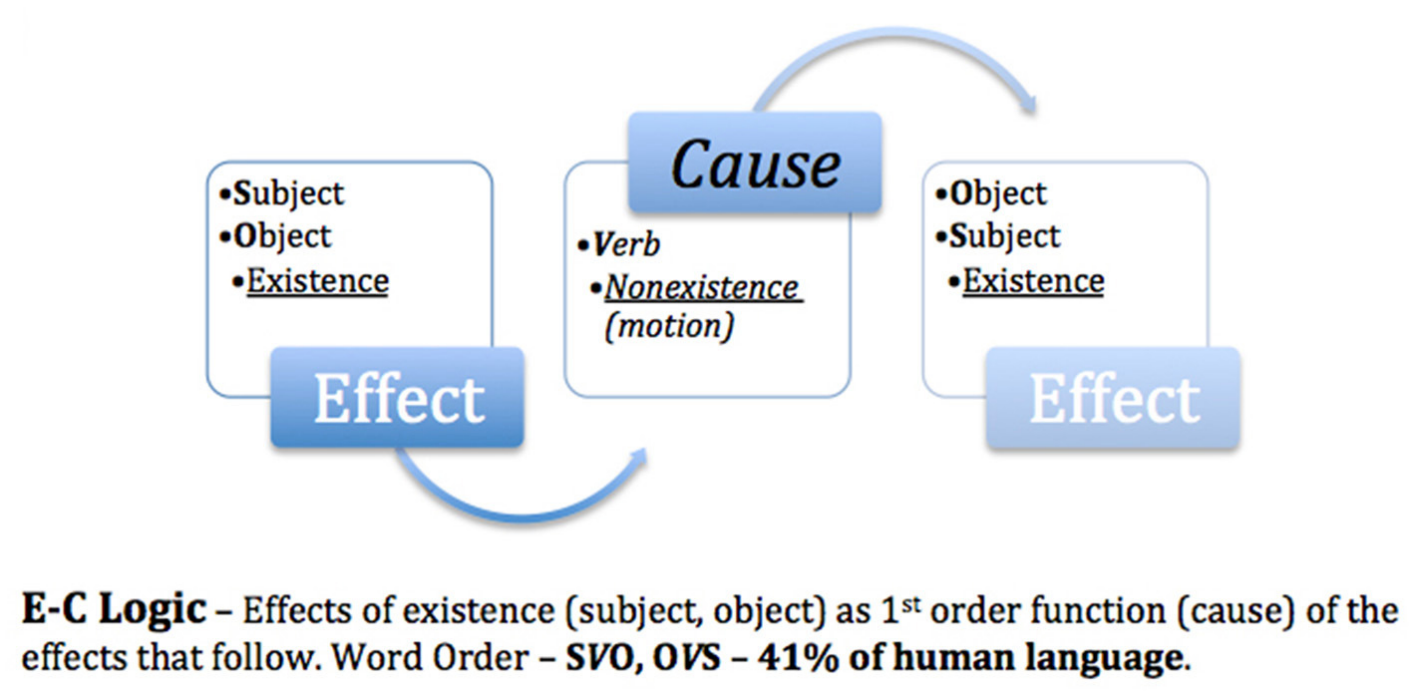
Effect causes effect.

**Figure 3:**
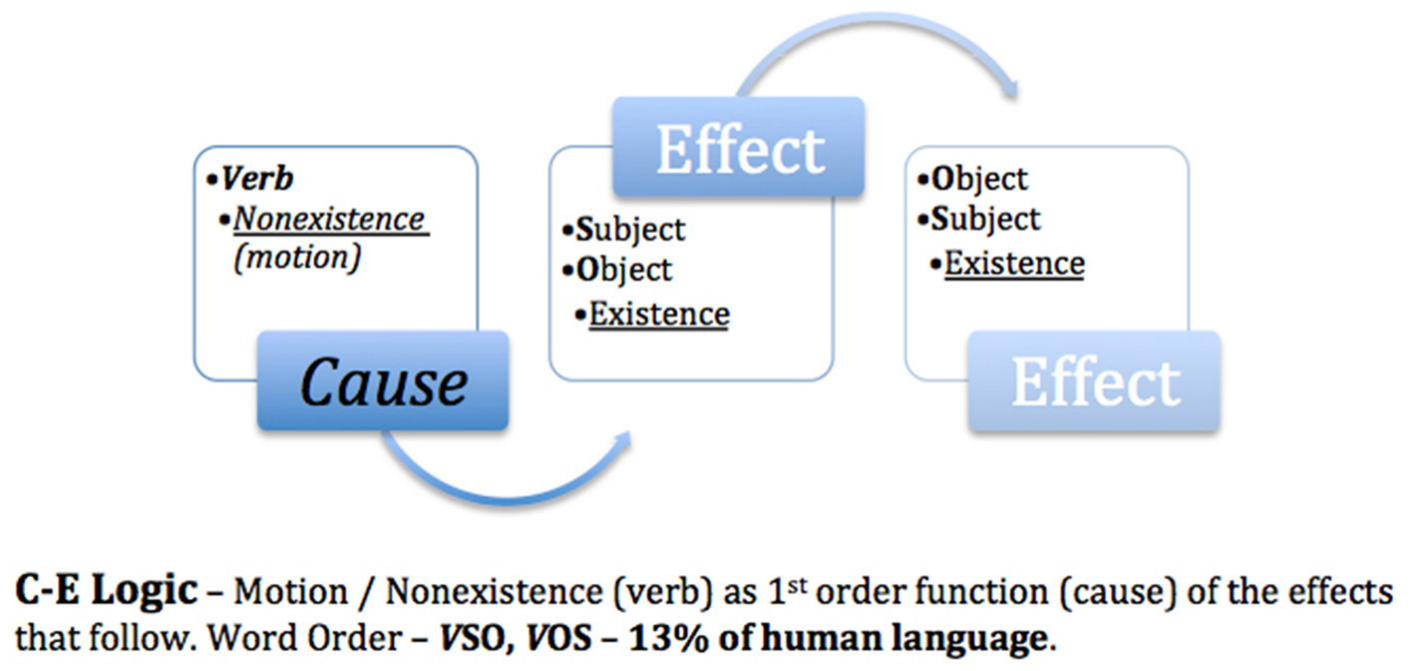
Cause precedes effects.

**Figure 4:**
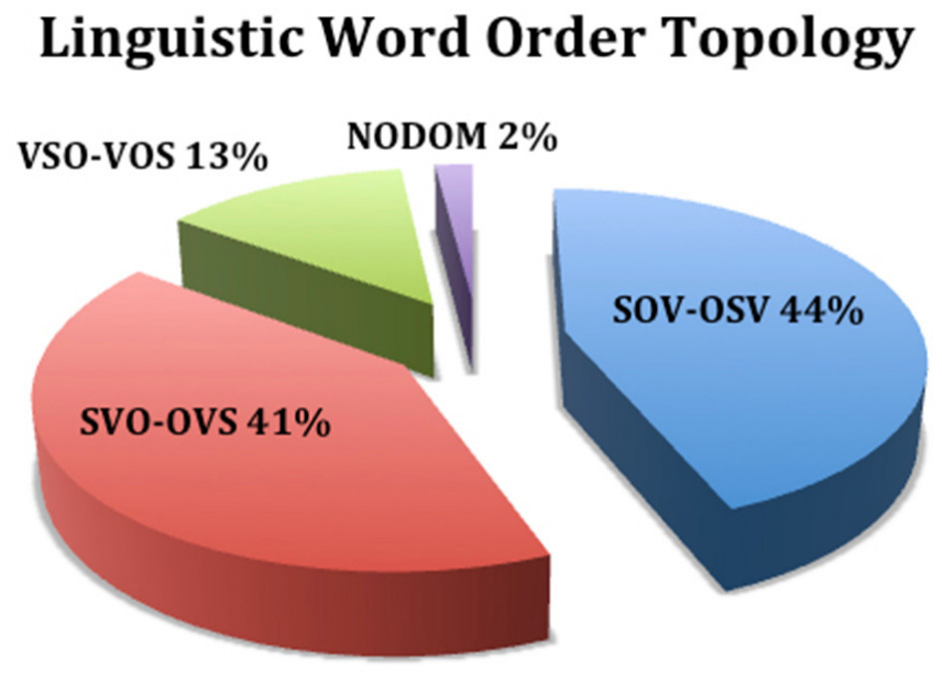
Dominant logic codes.

Returning to the initial question of “*How do we know what we think we know?”*, it is necessary to analyze the logic code that the human brain uses to perceive the outside world and then compare it with the theories and methods used in systematic studies for the advancement of science. The postulate put forth is “*How we think (logic) is how we speak”*. In a linguistic study of 5,252 world languages (Hammarström, 2016), word order typology revealed that 85% of the world's languages placed the causal property of motion (verb) as a second- or third-order function (Figures 1, 2), what I previously referred to as **E-C** logic (**E**ffects of existence–subject/object–before **C**ause), while 13% placed motion as a first-order function (Figure 3), **C-E** logic (**C**ause–verb–before **E**ffects of existence), and the remaining 2% used a combination of both word orders (Figure 4).

*This means that the human brain uses locality (E-C logic) as a first-order causal function but also uses non-locality (C-E logic) as a first-order causal function. Here lies the paradoxical logic (EPR Paradox) of the current situation*.

Because science is based on E-C logic (local input–cause–local output) both in theory and in the methods used to test such theories, bias is unavoidable. I trust that this is why Nobel laureates Alain Aspect, Anton Zeilinger, and John Clauser failed to test the super-deterministic loophole of Bell's theorem. As exemplified in Figure 1, E-C logic displaces motion/cause as a third-order function, thereby transforming *effects as a causal function*. In Figure 2, E-C logic displaces motion/cause as a second-order function between the effects of existence, e.g., fundamental interactions aka fundamental forces (Britannica, The Editors of Encyclopaedia, 2003), thus also establishing effects as causal.

However, in Figure 3, C-E logic positions motion/cause as a first-order function; as such, the dualistic effects of motion (direct selection and indirect selection) are causal of the effects that follow. Note that the logic code used to conduct the non-local no-go Tempt Destiny Experiment was based on C-E logic (non-local input–cause–local output). As empirically confirmed and exemplified in Table 1, C-E logic is inclusive of both local and non-local domains and is necessarily *in their proper order*, thereby eliminating the causal functions of motion as hidden variables, as exhibited in Figures 1, 2. Although absolute internal validity has been obtained (Tables 2, 3), it is imperative to establish the external validity of which mutually exclusive logic code, E-C logic or C-E logic, aligns with the mechanics of nature.

### 6.1 Double-slit experiment evaluation

To replicate the Method of Everything as detailed in the previous manuscripts of the initial findings (Morales, 2011a) and of the final results (Morales, 2016), the experimenter would need to recognize how the two mutually exclusive and jointly exhaustive selection functions of motion will be used to conduct their experiment. For example, on page 4 and pages 6-7 of the initial findings (Morales, 2011a), I touched upon how Young's local double-slit experiment (Young, 1804) and the non-local Tempt Destiny Experiment are essentially of the same construct (Morales, 2020) such that both experiments consist of two mutually exclusive experiments. The key difference is that the Tempt Destiny Experiment uses the non-local functions of motion as an input mechanism, whereas Young's experiment uses a local beam of light as its input mechanism, for example:

- Experiment 1. A single-slit serves as a single local measurement (also known as direct selection);
- Experiment 2. A double-slit serves as multiple local measurements (also known as indirect selection).

**Figure 5:**
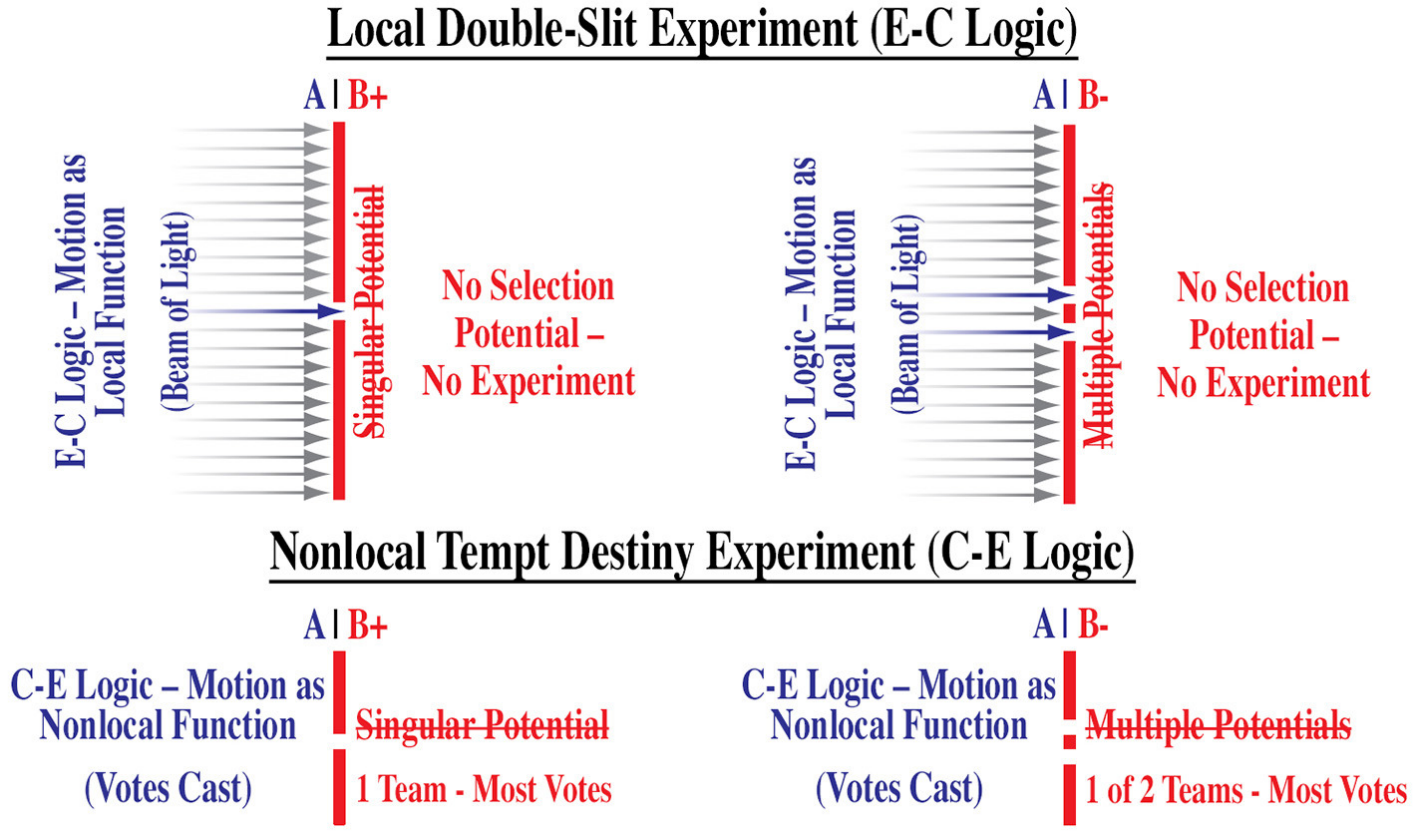
No act of motion, (A) no selection potentials, (B+, B-) no experiment.

Young's experiment consists of two mutually exclusive acts of measurement involving interactions between a local beam of light and two instruments of observation, i.e., a single-slit and a double-slit. As such, his experiment utilized the logic that *local existence is locally causal* such that the *effect* of, not the cause of, the local existence of a beam of light is understood to *cause* the local *effects* of his experiment, i.e., effect–cause–effect—E-C logic (local input–cause–local output). It should be noted that said effects demonstrated light as a particle (single-slit) and as a wave (double-slit), thus wave–particle duality serves as evidence of two mutually exclusive causal functions of two mutually exclusive effects (Britannica, The Editors of Encyclopaedia, 2024). When we understand that the two non-local functions of motion, direct selection (singular potential) and indirect selection (multiple potentials), are causal of local effects, such as measurement or observation (see Figure 5), we can then understand that the local effect of a wave function is the effect of an indirect selection, and the local effect of a particle function is an effect of a direct selection. Therefore, since both functions are mutually exclusive when a direct selection is applied to a wave function, it is predetermined to collapse to a particle function. It is from Young's experiments that the theory of quantum mechanics was derived and tested via the Bell-type experiments.

However, the Bell-type experiments and other indirect selection experiments do not use both causal functions to conduct their local experiments, e.g., LHC experiments, thus making their experiments incomplete. For example, instead of using double-slits, beam splitters were used to generate multiple output effects. They did not use the single local input of the photon beam to generate a single local output. The experimenters failed to recognize that in nature, as exhibited by Young's single-slit and double-slit experiments, there are two mutually exclusive and jointly exhaustive necessary causal functions. This omission error and the assumption that local existence is self-causal (E-C logic) has led to misinterpretations such as the wavefunction collapse, quantum entanglement, and Bell inequalities.

### 6.2 The Method of Everything evaluation

As mentioned in the previous manuscripts (Morales, 2011a, 2016), the non-local no-go Tempt Destiny Experiment tested whether the choice is determined by the experimenter or a predetermined function of a “super-deterministic” universe. The experiment came from repeating what had been performed twice before:

1) Cause: Each time a choice was made to support the NY Giants' Super Bowl quest;
2) Effect: A painting was created to be completed after the said team won the Super Bowl.

*(The unfinished painting procedure established a direct correlation of which mutually exclusive selection variable caused a certain or uncertain mutually exclusive effect; see* *Table 3*^†^*)*.

This event occurred in two of two previous attempts (see News 12 video). (Bergin, 2008) To test if the choice is a predetermined function, I invited football fans from all over the world to vote online in support of their team from 2000 to 2012. Before the conference championship games, the non-local acts of motion (votes cast) were then paired with the non-local potential of the team with the most votes also competing in the Super Bowl. In the first year of the experiment, it was confirmed that the mechanism we think of as choice is a dichotomy predetermined by nature, not determined by the experimenter (fans). Although acts of motion were obtained, the potential to become a selection was canceled, as exhibited in Tables 2, 3. Note that in the last 3 years of the 12-year Tempt Destiny Experiment, if a direct selection event failed to occur, then an indirect selection consisting of acts of motion paired with more than one potential, i.e., one of two teams competing in the Super Bowl (SB), was the indirect choice.

As exhibited in Table 1, the non-local no-go Tempt Destiny Experiment essentially consists of four experiments in their proper order: Part (1) Two mutually exclusive non-local experiments (cause) and Part (2) Two mutually exclusive local experiments (effects):

1) Non-local cause:

a. Direct Selection Experiment—Non-local input: Act of motion (votes cast) paired with a single potential (one team going to the Super Bowl);
b. Indirect Selection Experiment—Non-local input: Act of motion (votes cast) paired with multiple potentials (one of two teams going to the Super Bowl).

2) Local effects:

a. Direct Selection Experiment—Local output: Direct correlation between single non-local input and single local output—single effect/certain completion (SB XLII-W);
b. Indirect Selection Experiment—Local output: Direct correlation between multiple non-local inputs and multiple local outputs—multiple effects/uncertain completion (SB XLIV-W, SB XLV-L) ^*^. The SB XLVI painting's local potential was canceled by the team (Morales, 2016, p. 87) ^**^.

^*^The final effect of the potential completion of the artwork as caused by an SB win (W) (Morales, *2016**, Tables 1, 2, p. 87) was directly correlated to the two mutually exclusive and jointly exhaustive selection functions, thus eliminating ambiguity or need for statistical inference*.

As exhibited in Figure 5, the act of motion (A) is distinguished as a local or non-local function determined by the locality or non-locality of two mutually exclusive and jointly exhaustive selection potentials. In Young's local experiments, the beam of light (A) traveling through a single-slit (B+, single potential) or double-slit (B-, multiple potentials) would have been nullified if said local potentials (slits) were non-existent akin to the previously noted ^**^cancellation of the local potential of the SB XLVI painting. In the Tempt Destiny Experiment, the nullification of non-local potentials (Table 2) took place when the team with the most votes failed to compete in the Super Bowl. Regardless of the domain priority, local selection potentials (E-C logic) and non-local selection potentials (C-E logic) are necessary functions for a selection event. However, without motion selection, potentials are null.

*Cause as primary:* It is important to recognize that although the local potential effect of a completed SB XLVI painting had been negated, the non-local function of the indirect selection event needed to have first taken place. As such, cause is a non-local function, not a local effect of a wave function; subsequently, causation is not reversible. This means the mechanics are such that the local existence of the experimenter is not free to choose what nature has predetermined. *Therefore, all local experiments are determined by how they are predetermined by the non-local initial conditions of motion*.

## 7 The Method of Everything ≤ 1 inequality

Previously, I mentioned that obtaining a violation of Bell's theorem ( ≤ 2) via CHSH inequalities (2.8) would correspond with obtaining false-positive indirect selection results, as exhibited in Table 3. It is important to note that CHSH inequality is a mathematical construct that deals with classical correlations and, as such, is not specific to quantum theory. In the local Bell-type experiments, the choice is a local non-exclusive preexisting freedom (E-C logic). In the Tempt Destiny Experiment, choice is a non-local mutually exclusive mechanism that can only *come to exist* not preexist (C-E logic). Nonetheless, if we assume that both non-local selection functions preexist as assumed in the Bell-type experiments, then there would have been 24 choices made, 12 Alice (x, y) choices (DS) and 12 Bob (x, y) choices (IS) with two outcome/values, ≤ 2. Herein, let us analyze both logic codes against the evidence:

1) 12 Direct Selection (DS) potential attempts = 1 successful selection (one pos. potential outcome).
2) 12 Indirect Selection (IS) ^*^ potential attempts = 12 successful selections (two pos. potential outcomes).

^*^In the last 3 years of the TD experiment, three IS events occurred. If they were attempted for all 12 experiments, there would have been 12 successful attempts, including 1 false-positive DS attempt.

**Figure 6:**
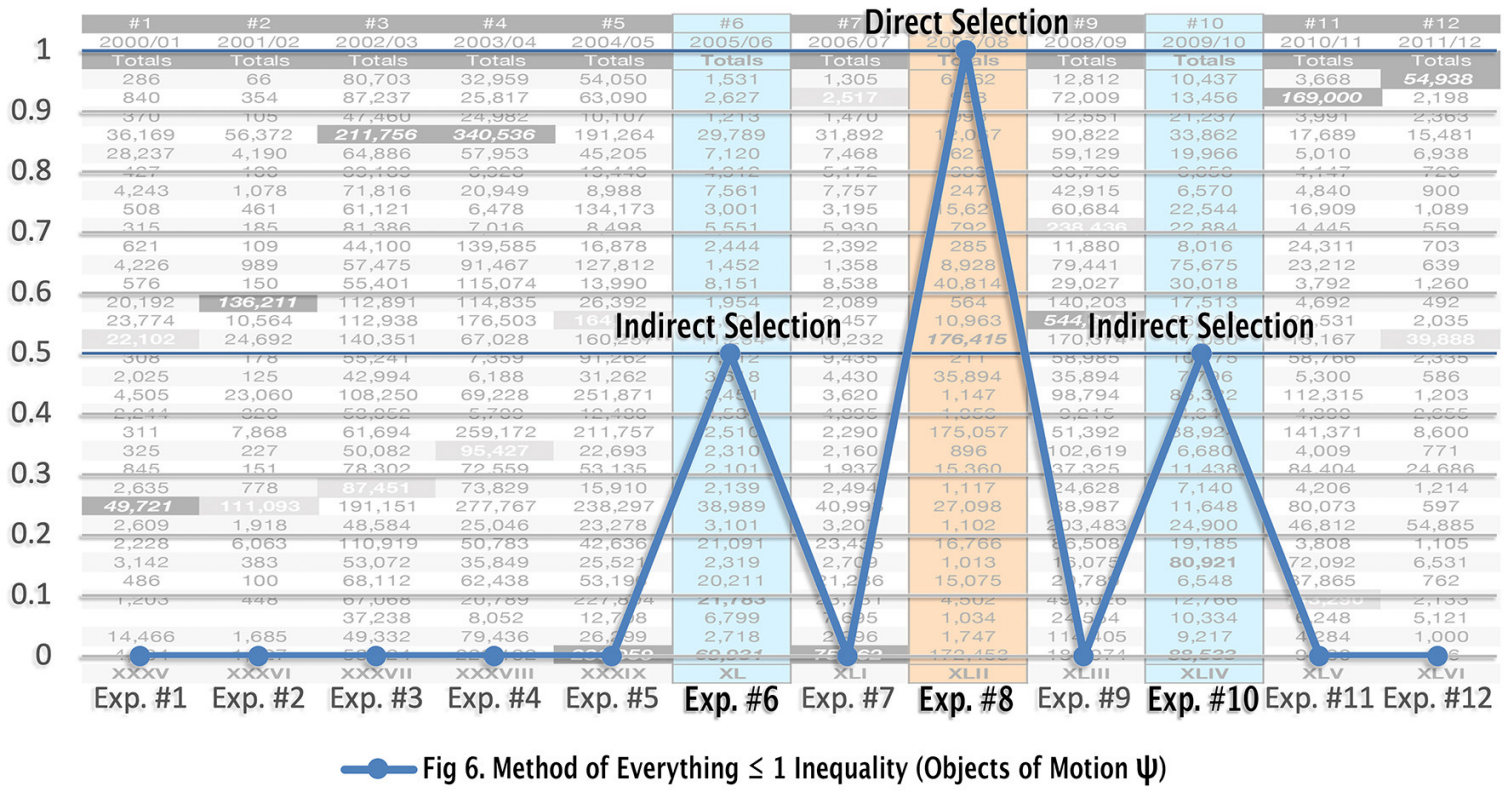
Wave function of human activity.

The combined number of 24 potential attempts yielded 3 positive potential completions of the unfinished artwork, 1-DS and 2-IS (Table 3† – 1-FP, 2-FN). The Method of Everything inequality of 24/3 = 8, which yields unequal value. The CHSH inequality includes two mutually exclusive causal functions as one function ( ≤ 2); thus, we get a false-positive value of 2.8. However, when we separate the two mutually exclusive outcomes/values of the Tempt Destiny Experiment as ≤ 1 (Figure 6), we then get a (2-IS) calculation of 12/2 = 6 = 0.5 value (50/50 split - double-slit) and a (1-DS) calculation of 12/1 = 12 = 1 value (single-slit), thus nullifying the 2.8 inequality since the *direct correlation* between two mutually exclusive and jointly exhaustive causal functions followed by two mutually exclusive effects are of equal values. As such, the Method of Everything ≤ 1 inequality threshold is not violated.

Therefore, to test if the non-local functions of motion do indeed supersede and thus cause local effects of existence as empirically confirmed in the Tempt Destiny Experiment, it is necessary for nature, not opinions, theories, or beliefs of man, to be the final arbitrator of which logic code abides by its laws.

## 8 The Method of Everything test

The ability to select, also known as choice, is assumed to be a cognitive function (intelligence) of a sentient being. Therefore, as a sentient entity governed by the laws of nature, the reader can use the effect of one's local existence to test if said *effect causes motion* (E-C logic) by removing the functions of motion from one's existence and thereby test if *motion caused the effect* of one's existence (C-E logic) or not. Note that since the experiment is based on the predetermined laws of nature, it is not recommended to conduct this experiment in real life. Nonetheless, this can be safely conducted as a logic experiment.

**The Final Selection Experiment** (Morales, 2021a,b)

You wake up one morning and find yourself completely paralyzed. This means that one cannot *directly select* to talk, eat food, drink fluids, go to the bathroom, etc., nor can anyone else *indirectly select* for one:

Can you *continue* your sentient existence without the non-local functions of motion?

The experimental results are self-evident, unambiguous, and universal. As an object of motion, *the mechanics are such* that the experimenter (mankind) cannot continue said existence without the source of one's existence. Therefore, as an initial condition (cause), the domain of motion supersedes the domain of existence (C-E logic), and because there are two mutually exclusive predetermined non-local functions of motion necessary to conduct all local experiments, failure to account for both functions in their proper order leads to erroneous scientific data (hidden variables), as shown in Table 3†. Therefore, it is imperative to address the experimenter bias issue of using E-C logic to conduct local experiments by using C-E logic to advance science to be a complete study of nature.

## 9 Conclusion

This bears repeating: nature, not the experimenter, dictates what can be tested but, more importantly, predetermines the necessary mechanics of how such tests can be executed.

By applying the Method of Everything inequality of ≤ 1 to the empirical evidence obtained in the Tempt Destiny Experiment (see Section 7.3), a wavelike property was revealed, as exhibited in Figure 6. The local effect of a wave function derived from objects of motion is congruent with the findings. For example, the external validity of the findings from the Tempt Destiny Experiment via the Final Selection Experiment establishes that human beings are, in essence, *objects of motion*. Therefore, data that exhibits effects of motion, e.g., a wave function, is to be expected. This means that regardless of scale, i.e., microscopic or macroscopic, states of existence are not causal of the effects of motion but are instead reflections of the effects of motion. Therefore, motion is causal (see Table 2) in the effects of existence such as the effect of a wave function. If one wishes to contest this claim, one can take up their argument with the predetermined laws of nature by simply conducting the Final Selection Experiment in real life. As a product of nature, I, for one, am not that arrogant or imprudent.

Note that the Method of Everything datasheet (Figure 6) revealed the ≤ 1 inequality boundary of 12 consecutive mutually exclusive events in an even-spaced sequential frequency (wave function) of 1 indirect selection event (0.5 value)−1 direct selection event (1 value)−1 indirect selection event (0.5 value). Direct correlation between two mutually exclusive and jointly exhaustive causal functions and their effects provided unambiguous empirical evidence of two mutually exclusive states, certainty and uncertainty. With said parameters empirically established, the evidence of two mutually exclusive and jointly exhaustive functions of motion supports Niels Bohr's complementarity principle (Bohr, 1937), inclusive of Werner Heisenberg's uncertainty principle and de Broglie-Schrödinger wavefunction duality (Heisenberg and Eckart, 1930); such that objects holding two complementary properties cannot be simultaneously observed or measured. However, the evidence also confirms Albert Einstein's conviction that quantum mechanics is incomplete (Einstein et al., 1935). In essence, what makes quantum mechanics incomplete is that it tells only half the story of the duplexity of Young's experiment. As previously noted, quantum mechanics is derived from the local wave/particle effects of the double-slits (indirect selection). However, it is not inclusive of the mutually exclusive local effect of the single-slit (direct selection). Failure to include and delineate the local effects of both mutually exclusive selection mechanisms explains why quantum mechanics is incomplete.

As previously stated, the local double-slit experiment and the non-local Tempt Destiny Experiment are similar since they both consist of two mutually exclusive experiments. However, with the inclusion of the mutually exclusive functions of the non-local domain of motion, the Tempt Destiny Experiment consists of four experiments—two non-local experiments directly correlated with two local experiments. By understanding that mankind is an object of motion, the activities of human beings emulating the effect of a wave function, as exhibited in Figure 6, are not only suitable but also contestable via the Final Selection Experiment.

In the manuscript titled “Spin States of Selection: Predetermined Variables of bit” (Morales, 2013), I graphed how the functions of selection can be otherwise understood as gravity. By understanding that the primary attraction of motion (G) with its potential functions (^2^) is of the first order, we can then understand how the measurable effect we call energy (something) came from nothing (attraction of motion with potential functions), E = G^2^. In other words, gravity (attraction) can be understood as a first-order function that causes the effects of angular momentum and linear momentum (see Supplementary material: Gravity - the Fundamental Force of Nature - E = G^2^). In addition, since the effects of motion cannot exist or preexist, this non-local function helps to explain how objects of motion evolve from one state of existence to another, which is what I call Choice/Chance Mechanics (Morales, 2011a).

For all local empirical studies, the discovery of non-local hidden variables necessitates a paradigm shift to account for variables that are predetermined to only *come to exist*. Since all local experiments have their effects, this means that *all experiments are valid expressions of said variables*. The assumption that a super-deterministic universe would discredit the validity of science, as noted by the Nobel laureates mentioned, is unjustified. To eliminate false-positive and false-negative empirical evidence, it is necessary to first establish which selection mechanism will be used to conduct a local experiment, knowing that direct selection experiments generate false-negative results (Table 3) and indirect selection experiments generate false-positive results. The same categorization is required for past experiments [subject of future discussion and analysis; see subsequent Non Disclosure Agreement (NDA) comment]. By changing the logic code we currently use to study nature (E-C logic), we can now use the logic code (C-E logic) that coincides with how nature works instead of insisting on how we want nature to be understood.

In the age of artificial intelligence, it is paramount to understand the current situation at hand. The Final Selection Experiment empirically invalidates the assumption that the non-local variables of selection are cognitive functions of a sentient being. Instead, what we think of as choice and or intelligence are predetermined non-local mechanical functions of nature. In other words, our ignorance of how nature attains something (existence) from nothing (motion) has unwittingly led to the creation of a new species also known as “artificial intelligence” (Novelli et al., 2023).

There is much more to discuss regarding how to apply the discovery of the non-local hidden variables of motion to study nature and safeguard mankind from an entity of our creation. Based on the fundamental mechanics involved, I conclude with the following suggestion. The reader's existence has helped provide absolute external validity of the Tempt Destiny Experiment such that all local empirical evidence is predetermined by one of the two mutually exclusive non-local selection functions. Subsequently, this means that all local data are generated accordingly. Therefore, all observable data, including Big Data (Leonelli, 2020), must also be predetermined by direct selection and indirect selection functions.

As a proof of concept, I had the opportunity to analyze the big data of a Fortune 50 Company (NDA prevents details) with the goal of establishing the key performance indicators (Parmenter, 2019) of their annual data. The multiple sources (companies) of the enormous amount of data generated could conceivably be compared to several LHC experiments running consecutively every day of the year. Regardless of the quantity and multiple input sources, I was able to distinguish the two mutually exclusive selection functions as key performance indicators of all their data. This should come as no surprise; in a super-deterministic universe, their data are predetermined by *how* they are determined.

## Data availability statement

The original contributions presented in the study are included in the article/Supplementary material, further inquiries can be directed to the corresponding author.

## Author contributions

MM: Conceptualization, Data curation, Formal analysis, Investigation, Methodology, Project administration, Resources, Supervision, Validation, Visualization, Writing – original draft, Writing – review & editing.

## Supplementary material

Data Sheet 1 QUEST 2023 Schedule.

Data Sheet 2 Tempt Destiny Experiment Data.

Data Sheet 3 Gravity – the Fundamental Force of Nature.

Image 1 E = G^2^.
