# Supplementary material for: The Method of Everything vs. Experimenter Bias of Loophole-Free Bell Experiments
Source: Front Res Metr Anal. 2024 Jul 11;9:1404371. doi: 10.3389/frma.2024.1404371 (PMC11269139; doi:10.3389/frma.2024.1404371)
Supplement: Data Sheet 1 — QUEST 2023 Schedule. [file Data_Sheet_1.PDF]

QUEST 2023

International Conference on

Quantum Engineered Sensing and Information Technology

June 27-30, 2023

Hotel Mercure Paris 19 Philharmonie-La Villette, 216 Av. Jean Jaures, 75019 Paris, France

Website: https://quest-conference.com/

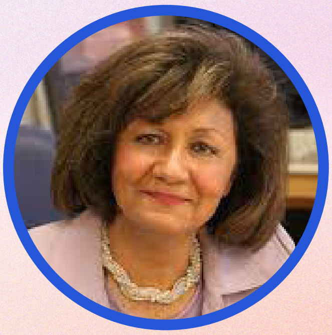

CHAIR PERSON

Prof. Manijeh Razeghi

Benjamin Franklin Award

(2018)

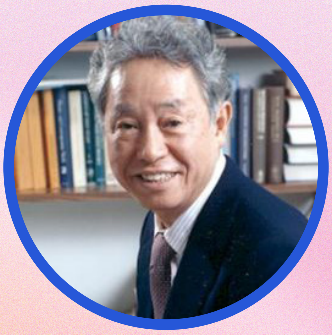

HONORARY CHAIR PERSON

Prof. Leo Esaki

Nobel Prize in Physics

(1973)

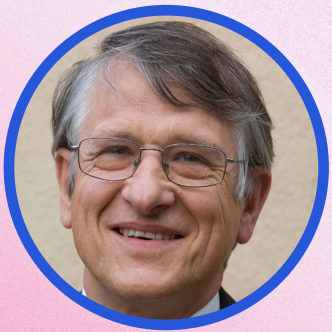

HONORARY CHAIR PERSON

Prof. Klaus Von Klitzing

Nobel Prize in Physics

(1985)

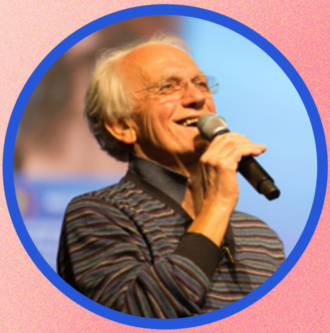

Prof. Gerard Mourou

Nobel Prize in Physics

(2018)

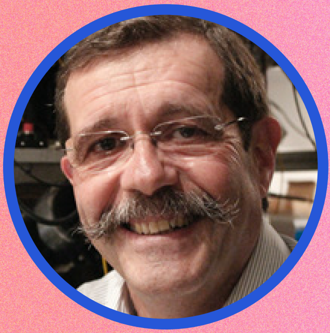

Prof. Alain Aspect

Nobel Prize in Physics

(2022)

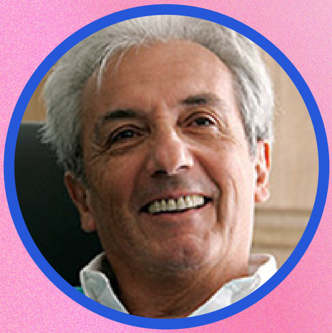

Prof. Albert Fert

Nobel Prize in Physics

(2007)

Experimenter Bias of Loophole-Free Bell Experiments

Manuel S Morales © 2023
msm@prephysics.com
Science, Math, Technology Division, Rowan College at Burlington County
Adjunct Professor (retired), Mount Laurel, NJ, USA

Abstract:

The 2022 Nobel Prize in Physics is for "loophole-free" Bell experiments which provided empirical evidence that contradicts local realistic theories of nature and thereby validates quantum mechanics as a fundamental theory. The simultaneous closure of the locality loophole and the detection loophole of Bell's inequality theorem in 2022 constitute Bell experiments as loophole-free. However, unambiguous empirical evidence obtained in a twelve-year (2000-2012) selection-based experiment confirmed that choice, i.e., direct selection and indirect selection, are nonlocal (hidden) mechanisms of motion that can only come-to-exist not preexist or be existent. *The evidence reveals that what we think of as choice is a mechanical function predetermined by nature not a freedom determined by the experimenter*. The bias of experimenters not closing or wittingly ignoring the remaining "free-will" or "freedom-of-choice" loophole means that loophole-free Bell experiments are in fact not loophole-free. Furthermore, the assumption of using the construct of Bell experiments (indirect selection experiments) to close the freedom-of-choice loophole would necessitate experimenters do not use the nonlocal mechanism of indirect selection (choice) to conduct their experiments. In other words, it is impossible to close a predetermined function that can only come-to-exist not preexist or exist as a "freedom" or experimenter option – a claim that can be empirically confirmed without ambiguity via the Final Selection Experiment. The bias of ignoring the two mutually exclusive and jointly exhaustive nonlocal variables of motion necessary to conduct so-called loophole-free Bell experiments inherently leads to false-positive and false-negative empirical evidence. That being said, all empirical evidence has been created by the variables of motion. This means that the raw data in empirical investigations such as CERN's LHC experiments contain the hidden codes of the fundamental variables that created their data. Knowledge of the unambiguous codes will reveal algorithms that can be applied towards the advancement of science once the experimenter bias issue has been addressed.

Key Performance Indicators Reveal Hidden Origin Variables

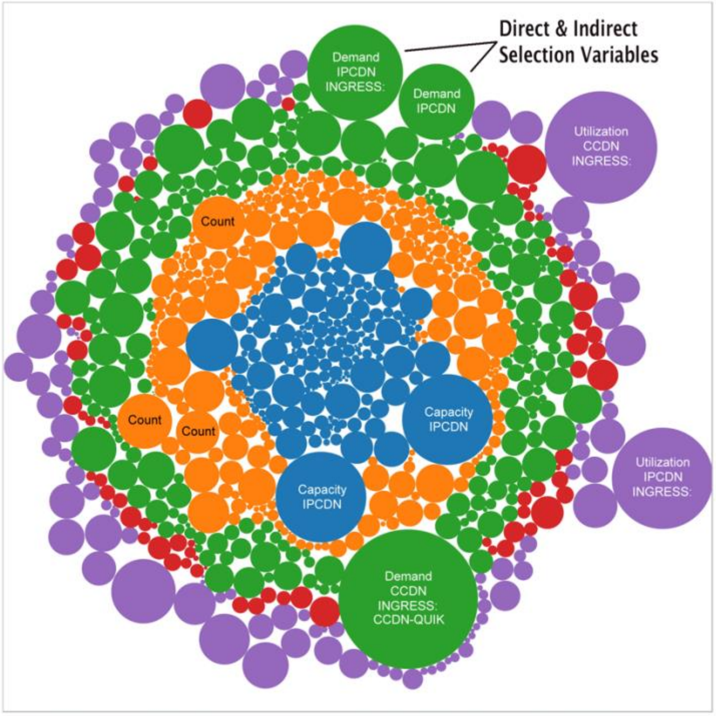

NOTE: Annual data generated 24/7/365 from over two dozen input sources consisting of hundreds of millions of interactions per second.

Biography of presenting author

Manuel S Morales is an independent researcher in the field of fundamental mechanics, i.e., origin physics. He has a BFA in illustration and AS in photography. His notable career as an artist inadvertently led to conducting a twelve-year nonlocal no-go experiment at TemptDestiny.com which obtained unambiguous empirical evidence of Einstein's (nonlocal) hidden variables. He has applied his findings to particle physics, theoretical physics, experimental physics, condensed matter physics, and data analysis of key performance indicators of a Fortune 50 company. In addition to published articles and lectures at physics conventions, he has also served as a referee for several physics journals.

Details of presenting author:

Name: Manuel S Morales
Affiliation: Rowan College at Burlington County
Country: USA

Other Details:

Presentation Category: Invited Speaker
Session Name: Breakthrough Scientific Results - https://quest-conference.com/speakers.php
Email: msm@prephysics.com
Alternative email: admin@temptdestiny.com
Research Website: http://TemptDestiny.com
Contact Number:
LinkedIn: https://www.linkedin.com/in/manuel-morales-421b882b/

Recent Photograph:

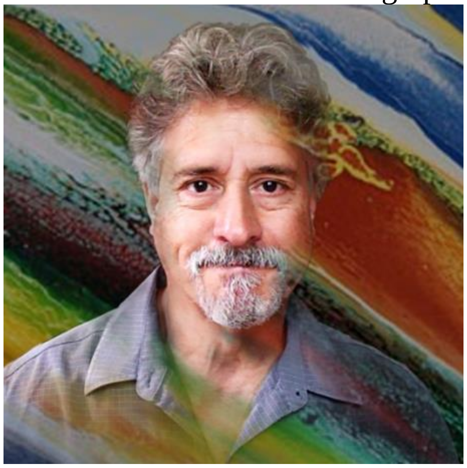

QUEST 2023

International Conference on Quantum Engineered Sensing and Information Technology

June 27-30, 2023| Paris, France

**Day-1 June 27, 2023**	
**Hall Name: ETOILE LOUVRE**	
08:00-08:30	**Onsite Registrations**
08:30-08:40	**Moderator Introduction**
08:40-09:00	**Opening Ceremony by Honorable Guests**
	**Plenary Session**
09:00-09:20	***Introduction***
	**Prof. P.L Meunier, Prof. J.P Huignard, Prof. M. Razeghi**
09:20-09:40	**Prof. Jerzy M. Langer,** Warsaw Scientific Society, Poland
	**Title:** European Innovation Council -A star in the making
09:40-10:00	**Dr. Bernhard Quendt,** Chief Technical Officer, Thales, France
	**Title:** The second Quantum Revolution from an industrial Perspective
10:00-10:30	**Coffee Break@ TROCADERO**
10:30-10:40	**1981-1991 LCR Thomson CSF FRANCE**
	**Dr Olivier Acher , Dr DEFOUR Martin, Dr. J.P Huignard, Prof. P.L Meunier**
	**1993-2023 CQD Northwestern University USA**
10:40-10:50	**Prof. Steve Silkin, Prof. Hooman Mohseni, Dr. Nguyen Binh-Minh , Prof. Pedram Khallili**
	**2022-2023 Award Winners**
10:50-11:00	**Prof. Alain Aspect, Prof. Gérard MOUROU, Prof. Nader Engheta, Prof. Dariush Afshin**
11-00-13.00	**Group photo & Lunch @ PANORAMIC**
**Session 1:**	**Session chaire: Prof. Dr. Elaesser Wolfgang, Prof Fainman Yeshaiahu**
13:30-14:00	**Dr. Dominique M. Dagenais,** National Science Foundation, U.S.A
	**Title:** The Quantum Leap Program at the National Science Foundation
14:00-14:20	**Prof. Helmy Amr S,** Univeristy of Toronto, Canada
	**Title:** Semiconductor Circuits for Quantum Enhanced LIDAR Systems
14:20-14:40	**Prof. Mehdi Alouini,** Institut Foton, UNIVREN/CNRS, Rennes, France
	**Title:** Ultra-narrow linewidth self-adaptive photonic oscillator: From principle to product
14:40-15:00	**Prof. Friedman Joseph,** The University of Texas at Dallas, USA
	**Title:** Spintronic Phenomena for Reversible, Neuromorphic, and Reservoir Computing
15:00-15:30	**Coffee Break@ TROCADERO**
**Session 2**	**Session chaire: Prof. Masud Mansuripur, Prof Fabrice DEVAUX**
15:30- 15:50	**Dr. Andrew Thain,** Space Systems, AIRBUS, Toulouse, France
	**Title:** Space based quantum communications at Airbus
15:50-16:10	**Dr Henri Jaffres,** Université Paris-Saclay, F-91767 Palaiseau, France
	**Title:** Ultrafast spin-charge conversion in topological insulators surface states probed by THz emission spectroscopy

16:10- 16:30	**Dr. Tonouchi Masayoshi,** Osaka University, Japan
	**Title:** Terahertz Emission Spectroscopy and Imaging of Semiconductor Heterostructures and Quantum Wells
16:30- 16:50	**Prof. Ali Adibi,** Georgia Institute of Technology, USA
	**Title:** Phase-change Materials for Reconfigurable Metaphotonics
16:50- 17:10	**Prof. Jonas LARSON,** Stockholm University, Sweden
	**Title:** Quantum optics in state space
17:10- 17:30	**Prof. Pereira Mauro F,** Khalifa University of Science and Technology, UAE
	**Title:** Giant Control of GHz-THz Nonlinearities in Semiconductor Superlattices
17:30- 17:50	**Prof. Johannes Kunsch,** Laser Components Germany GmbH, Germany
	**Title:** Updates on selected classic infrared components for benchmarking: PbS, PbSe, DLaTGS and LiTaO3 detectors
**Day-2 June 28, 2023**	
**Hall Name: ETOILE LOUVRE**	
**Session 3**	**Session chaire: Dr. Bernanhard QUENDT, Prof Fabien Bretenaker**
	**Plenary Talk**
**08:30-09:00**	**Prof. Alber Fert,** Unité Mixte de Physique CNRS-Thales, Université Paris-Sud, Université Paris-Saclay, Palaiseau, France
	**Title:** Topological spintronics: From skyrmions to topological insulators
09:00- 09:20	**Prof. Wolfgang ELAESSER,** Technische Universitaet Darmstadt, Germany
	**Title:** Quantum Sensing with Photon Correlations of Classical Light: Ghost Imaging, Ghost Spectroscopy and Ghost Polarimetry
09:20-09:40	**Dr. Taichi OTSUJI,** Tohoku University, Japan
	**Title:** Graphene-based 2D Heterostructures for Plasmonic Terahertz Laser Transistors and Detectors
09:40-10:00	**Prof Giti KHODAPARAST,** Virginia tech, USA
	**Title:** Optical Probe of Coherent States in Multi-Functional Materials
**10:00-10:30**	**Coffee Break@ TROCADERO**
**Session 4**	**Session chaire: Prof Hooman MOHSENI, Prof. Helmy Amr S**
10:30-10:50	**Prof AGNES MAITRE,** Sorbonne Université, France
	**Title:** Dramatic acceleration and spectral broadening of Cdse /CdS single nanocrystal emission under high excitation or large confinement
10:50-11:10	**Prof Pedram Khalili,** Northwestern University, USA
	**Title:** Spin-orbit torque switching of metallic antiferromagnets and ferrimagnets
11:10- 11:30	**Prof. Yossi PALTIEL,** The Hebrew University, Israel
	**Title:** Chiral molecules and the electron spin
11:30-11:50	**Dr. Olivier Acher,** HORIBA France SAS, Palaiseau, France
	**Title:** An in-plane position sensing technique with nm resolution based on machine vision: application to microscopy and laboratory activities
11:50-12:10	**Prof. Igor ZUTIC,** University at Buffalo, USA
	**Title:** Proximitized Quantum Materials: From Superconducting Spintronics to Majorana States
**12:10-13:10**	**Lunch @PANORAMIC**
**Session 5**	**Session chaire: Prof Nader ENGHETA, Dr. Jean-Pierre HUIGNARD**
**13:30-14:00**	**Prof. Eli Yablonovitch,** University of California, USA
	**Title:** Optical Physics Does Digital Optimization—which we call Onsager Computing—for Machine Learning, Control Theory, Backpropagation, etc.

14:00-14:20	**Prof. Demetri PSALTIS,** Ecole Polytechnique Federale de Lausanne (EPFL), Switzerland
	**Title:** Programming optical learning machines
14:20- 14:40	**Prof. Christophe MOSER,** Ecole Polytechnique Federale de Lausanne (EPFL), Switzerland
	**Title:** Nonlinear Processing with Only Linear Optics (nPOLO)
14:40-15:00	**Prof Masud Mansuripur,** The University of Arizona, USA
	**Title:** Fundamental properties of beam-splitters in classical and quantum optics
**15:00-15:30**	**Coffee Break@ TROCADERO**
**Session 6**	**Session chaire: Prof Selim Shahriar , Dr. Martin DEFOUR**
15:30-15:50	**Prof. Frederic Grillot,** Institut Polytechnique de Paris, France
	**Title:** Secured free-space optics with mid-infrared quantum cascade lasers
15;50- 16.10	**Prof. Yong Hang ZHANG,** Arizona State University, USA
	**Title:** InAs/InAsSb type-II superlattice: Its material properties and applications in IR lasers and photodetectors
16;10-16.30	**Dr. Thierry DEBUISSCHER,** Thales Research & Technology, France
	**Title:** Quantum sensing with ensembles of NV centers in diamond
16.30-16.50	**Prof. Amaud LANDRAGIN,** Sorbonne Université, France
	**Title:** Quantum sensors with atomic interferometry
16.50-17;10	**Dr. Marina YAKOVLEVA,** Université Paris-Saclay, France
	**Title:** Perfect coupling conditions for MIM antenna in zero magnetic field regions
**Day-3 June 29, 2023**	
**Hall Name: ETOILE LOUVRE**	
**Session 7**	**Session chaire: Prof Christos FLYTZANIS, Prof Jerzy M. LANGER**
	**Plenary Talk**
**08:30-09:00**	**Prof. Alain ASPECT,** Institut d'Optique Graduate School / Université Paris-Saclay École Polytechnique / Institut Polytechnique de Paris, France
	**Title:** Single Photons, Entangled Photons: From Quantum Foundations to Quantum Technologies
09:00-09:20	**Prof. Fabrice DEVAUX,** Université Bourgogne Franche-Comté15b, France
	**Title:** Two-photon holography and interference in twin images at the quantum level
09:20-09:40	**Dr. Fabien BRETENAKER,** Université Paris-Saclay, ENS Paris-Saclay, CentraleSupélec, CNRS, France
	**Title:** Quantum Optics in a Metastable Helium Vapor
09:40-10:00	**Prof. Manuel S. MORALES,** Rowan College at Burlington County, USA
	**Title:** Experimenter Bias of Loophole-Free Bell Experiments
**10:00-10:30**	**Coffee Break@ TROCADERO**
**Session 8**	**Session chaire: Prof Frederic GRILLOT, Prof Lars SAMUELSON**
10:30-10:50	**Dr. Murzy JHABVALA,** NASA Goddard Space Flight Center, USA
	**Title:** Overview of SLS-Based Instrument Development at NASA/Goddard Space Flight Center
10:50-11:10	**Prof. Selim SHAHRIAR,** Northwestern University, USA
	**Title:** Advanced Quantum Sensors: Superluminal Lasers, Subluminal Lasers and Schroedinger Cat Atomic Interferometers
11:10-11:30	**Dr. Maxime OLIVA,** Atos Quantum Lab, Atos, Les Clayes-Sous-Bois, Yvelines, France
	**Title:** Towards Fermionic Systems Simulations on Quantum Computers with Myqlm-Fermion
11:30-11:50	**Prof. Rui Q. Yang,** University of Oklahoma, USA
	**Title:** Interband Cascade Infrared Photodetectors for High Temperature and High-Speed Operation

11:50-12:10	**Dr. Matthieu Dupont -Nivet,** Thales Resaerch and Technology, France
	**Title:** Inertial Navigation with cold atom on chip
**12:10-13:10**	**Lunch @PANORAMIC**
**Session 9**	**Session chaire: Dr. Ito HIROSHI, Prof Eli Yablonovitch**
**13:30-14:00**	**Prof. Nader Engheta,** University of Pennsylvania, USA
	**Title:** Structuring Light with Metastructures
14:00-14:20	**Dr. Yannick de WILDE,** ESPCI Paris, PSL University, CNRS, Institut Langevin, France
	**Title:** Probing the infrared thermal radiation of patch antennas
14:20- 14:40	**Dr. Nils C. Gerhardt,** Ruhr-University Bochum, Germany
	**Title:** Spin-Lasers: With Ultrafast Polarization Modulation to the Next Generation of Optical Communication Systems
14:40-15:00	**Dr. John PRINEAS,** University of Iowa, USA
	**Title:** Purcell Effect Versus Auger Scattering in Resonant Mid-Infrared W-Superlattice LEDs
**15:00-15:30**	**Coffee Break@ TROCADERO**
**Session 10**	**Session chaire: Dr. Maxime OLIVA, Dr. Yannick de WILDE**
15:30-15:50	**Dr. Joseph Tischler,** University of Oklahoma, USA
	**Title:** Hyperbolic Phonon Polaritons as a Route for Nanophotonic Devices
15:50-16:10	**Prof. Jeong Woo HAN,** Universität Duisburg-Essen, Germany
	**Title:** Nonlinear THz absorption in graphene plasmons
16:10-16:30	**Dr. Mi ZETIAN,** University of Michigan, USA
	**Title:** Ferroelectric nitride semiconductors: Epitaxy, quantum engineering, and emerging applications
16:30-16:50	**Prof. Amanti Maria Ines,** Université Paris Diderot, France
	**Title:** Generation and manipulation of frequency states of light with AlGaAs quantum sources
16:50-17:10	**Prof. Hooman Mohseni,** Northwestern University, USA
	**Title:** Energy-Efficient Integrated Nano-Phototransistors
17:10-17:30	**Dr. Yoshie OTAKE,** RIKEN Center for Advanced Photonics, Japan
	**Title:** RIKEN Accelerator-driven compact neutron sources, RANS, and their applications
17:30-17:50	**Dr Samuelson Lars,** Lund University, Sweden
	**Title:** Realization of InGaN nanoLEDs delivering blue, green and red light
**Day-4 June 30, 2023**	
**Hall Name: ETOILE LOUVRE**	
**Session 11**	**Session chaire:Prof Paul-Louis MEUNIER, Dr. Ferechteh HOSSEINI- TEHERANI**
**08:30-09:00**	**Prof. Gérard MOUROU,** École polytechnique Palaiseau, France
	**Title:** Searching for Extreme Light
09:00-09:20	**Dr. Afshin DARYOUSH,** Drexel University, Philadelphia, USA
	**Title:** Ultra-Broadband Compact Frequency Synthesizers Using Self-Forced Multi-Mode Multi-Quantum Semiconductor Lasers
09:20-09:40	**Dr. Philippe LALANNE,** CNRS-IOGS-Univ Bordeaux, Bordeaux, France
	**Title:** Interaction of light with non-Hermitian plasmonic nanoresonators: The mode volume
09:40-10:00	**Prof. Leo GIUSEPPE,** Université de Paris - CNRS, Paris, France
	**Title:** Second harmonic generation with wavefront control on dielectric metasurfaces
**10:00-10:30**	**Coffee Break@ TROCADERO**

Session 12:	Session chaire: Dr. Afshin DARYOUSH, Prof Drouhin Henri-Jean
10:30-10:50	**Dr. Yeshaiahu (shaya) Fainman,** University of California San Diego, USA
	**Title:** Nanoscale Light Emitters and their Dinamics
10:50-11:10	**Dr. NGUYEN Binh-Minh,** HRL Laboratories, LLC, USA
	**Title:** Antimonide-based Narrow Bandgap Semiconductors for Infrared Technology and Quantum Information Science
11:10-11:30	**Dr. Jean-Francois GUILLEMOLES,** CNRS-Ecole Polytechnique/IPParis-ENSCP/PSL-IPVF/SAS 18 boulevard Thomas Gobert, France
	**Title:** Photovoltaic Conversion by Reciprocity
11:30-11:50	**Dr. Ito HIROSHI,** The University of Tokyo, Japan
	**Title:** Low-Noise Terahertz-Wave Detector: Fermi-Level Managed Barrier Diode
11:50-12:10	**Prof. Vijaysekhar Jayaraman,** Praevium Research, Inc., USA
	**Title:** Detectors and Emitters for Mid-Wave Infrared Optical Communications
**12:10-13:10**	**Lunch @PANORAMIC**
**Session 13:**	**Session chaire: Prof Pedram KHALLILI, Dr. Binh-Minh NGUYEN**
**13:30-14:00**	**Prof. Christos Flytzanis,** Ecole Normale Supérieure, France
	**Title:** Fashioning and Pumping up the Sound. Photodriven Coherent THz Acoustic Phonon Amplification and Quantum Cascade Saser Operation in Semiconductor Superlattices
14:00-14:10	**Dr. Luc DAME,** Laboratoire Atmosphères, Milieux, Observations Spatiales (LATMOS), IPSL/CNRS & University Paris-Saclay, France
	**Title:** New Disruptive Solar-blind UVC Sensors for New Space Applications
14:10-14:20	**Dr. Linda J. OLAFSEN,** Baylor University, USA
	**Title:** Quasi-Fermi Level Pinning and Optical Pumping Analysis toward Reduction of Droop in Interband Cascade Lasers
14:20-14:30	**Dr. Sebastien BIDAULT,** Institut Langevin, ESPCI Paris, Université PSL, CNRS, Paris, France
	**Title:** Purcell Effect in Plasmonic and Dielectric Resonators
14:30-14:40	**Dr. Mikhail NESTOKLON,** Ioffe Institute, Russia
	**Title:** Exciton Fine Structure in Lead Chalcogenide Quantum Dots: Interplay between Valley Mixing and Exchange Interaction
14:40-14:50	**Dr. Paola di PIETRO,** Elettra-Sincrotrone Trieste S. C. p. A., Italy
	**Title:** Nonlinear ultrafast studies on Dirac materials at TeraFERMI beamline
14:50-15:00	**Dr. David HEYDARI,** Stanford University, USA
	**Title:** Electric-field induced nonlinear optics in CMOS silicon nanophotonic waveguides
15:00-15:10	**Dr. Paolo ROCCHI,** IBM and LUISS University, Italy
	**Title:** An Engineering Theory of Sensing
15:10-15:20	**Prof. Remi LEANDRE,** Université de Bourgogne-Franche-Comté, France
	**Title:** Toward a Wong-Zakai approximation for big order generators
15:20-15:30	**Dr. Bertels KOEN,** University of Ghent and QBee.eu, Belgium
	**Title:** Quantum Computing Logic – an example for Quantum Genomics

Poster session Chairs; Prof Giti Khodaparast ,Prof Linda J. Olafsen	
QUEST P-01	**Dr. Szymon Tofil,** Kielce University of Technology, Poland
	Different Effects of Laser Removal of Coatings with Different Lasers Devices
QUEST P-02	**Dr. Kurp Piotr,** Kielce University of Technology, Poland
	Mechanically Assisted Laser Forming of New Kind Helical Metal Expansion Joints
QUEST P-03	**Dr. Leonard CARDINALE,** University of Oxford, Oxford, UK
	Nuclear Spin Control in GaAs Quantum Dots via Nuclear Quadrupole Resonance
**15.30-17.00 Conference Closing Ceremony and Group photo**<br>**Coffee Break@ TROCADERO**	
