# Supplementary material for: The Method of Everything vs. Experimenter Bias of Loophole-Free Bell Experiments
Source: Front Res Metr Anal. 2024 Jul 11;9:1404371. doi: 10.3389/frma.2024.1404371 (PMC11269139; doi:10.3389/frma.2024.1404371)
Supplement: Data Sheet 2 — Tempt Destiny Experiment Data. [file Data_Sheet_2.PDF]

Tempt Destiny Experiment Data © (2000-2012)

Experiment	#1	#2	#3	#4	#5	#6	#7	#8	#9	#10	#11	#12
NFL Season	2000/01	2001/02	2002/03	2003/04	2004/05	2005/06	2006/07	2007/08	2008/09	2009/10	2010/11	2011/12
NFL Teams	Totals	Totals	Totals	Totals	Totals	Totals	Totals	Totals	Totals	Totals	Totals	Totals
49ERS	286	66	80,703	32,959	54,050	1,531	1,305	6,562	12,812	10,437	3,668	***54,938***
BEARS	840	354	87,237	25,817	63,090	2,627	**2,517**	958	72,009	13,456	***169,000***	2,198
BENGALS	370	105	47,460	24,982	10,107	1,213	1,470	993	12,551	21,237	3,991	2,363
BILLS	36,169	56,372	***211,756***	***340,536***	191,264	29,789	31,892	12,067	90,822	33,862	17,689	15,481
BRONCOS	28,237	4,190	64,886	57,953	45,205	7,120	7,468	621	59,129	19,966	5,010	6,938
BROWNS	427	100	39,189	6,928	19,440	4,912	5,172	989	30,796	6,658	4,147	726
BUCCANEERS	4,243	1,078	71,816	20,949	8,988	7,561	7,757	247	42,915	6,570	4,840	900
CHARGERS	508	461	61,121	6,478	134,173	3,001	3,195	15,627	60,684	22,544	16,909	1,089
CARDINALS	315	185	81,386	7,016	8,498	5,551	5,930	792	**238,436**	22,884	4,445	559
CHIEFS	621	109	44,100	139,585	16,878	2,444	2,392	285	11,880	8,016	24,311	703
COLTS	4,226	989	57,475	91,467	127,812	1,452	1,358	8,928	79,441	75,675	23,212	639
COWBOYS	576	150	55,401	115,074	13,990	8,151	8,538	40,814	29,027	30,018	3,792	1,260
DOLPHINS	20,192	***136,211***	112,891	114,835	26,392	1,954	2,089	564	140,203	17,513	4,692	492
EAGLES	23,774	10,564	112,938	176,503	**164,964**	3,101	2,457	10,963	***544,615***	36,168	22,531	2,035
GIANTS	**22,102**	24,692	140,351	67,028	160,257	11,234	10,232	***176,415***	170,374	17,030	15,167	**39,888**
FALCONS	308	178	55,241	7,359	91,262	7,812	9,435	211	58,985	10,175	58,766	2,335
JAGUARS	2,025	125	42,994	6,188	31,262	3,618	4,430	35,894	35,894	7,796	5,300	586
JETS	4,505	23,060	108,250	69,228	251,871	3,451	3,620	1,147	98,794	86,322	112,315	1,203
LIONS	2,244	320	53,052	5,709	12,480	4,581	4,895	1,056	9,215	4,644	4,390	2,655
PACKERS	311	7,868	61,694	259,172	211,757	2,510	2,290	175,057	51,392	38,924	141,371	8,600
PANTHERS	325	227	50,082	**95,427**	22,693	2,310	2,160	896	102,619	6,680	4,009	771
PATRIOTS	845	151	78,302	72,559	53,135	2,101	1,937	15,360	37,325	11,438	84,404	24,686
RAIDERS	2,635	778	**87,451**	73,829	15,910	2,139	2,494	1,117	24,628	7,140	4,206	1,214
RAMS	***49,721***	**111,093**	191,151	277,767	238,297	38,989	40,993	27,098	88,987	11,648	80,073	597
RAVENS	2,609	1,918	48,584	25,046	23,278	3,101	3,207	1,102	203,483	24,900	46,812	54,885
REDSKINS	2,228	6,063	110,919	50,783	42,636	21,091	23,435	16,766	86,508	19,185	3,808	1,105
SAINTS	3,142	383	53,072	35,849	25,521	2,319	2,709	1,013	16,075	**80,921**	72,092	6,531
SEAHAWKS	486	100	68,112	62,438	53,196	20,211	21,286	15,075	20,789	6,548	37,865	762
STEELERS	1,203	448	67,068	20,789	227,894	**21,783**	23,731	4,502	498,026	12,766	**143,290**	2,133
TEXANS			37,238	8,052	12,798	6,799	7,695	1,034	24,554	10,334	6,248	5,121
TITANS	14,466	1,685	49,332	79,436	26,299	2,718	2,996	1,747	114,405	9,217	4,284	1,000
VIKINGS	4,084	1,127	58,724	220,192	***286,059***	***69,931***	***75,262***	172,453	181,974	***88,533***	9,980	466
**Super Bowl**	**XXXV**	**XXXVI**	**XXXVII**	**XXXVIII**	**XXXIX**	**XL**	**XLI**	**XLII**	**XLIII**	**XLIV**	**XLV**	**XLVI**
**TOTAL VOTES**	234,023	391,150	2,489,976	2,597,933	2,671,456	307,105	326,347	748,353	3,249,347	779,205	1,142,617	244,859
**15,182,371**												
**Selection Events (Cause)**								***Direct***		**Indirect**	**Indirect**	**Indirect**
False-Positive (1)	Indirect	Indirect	Indirect	Indirect	Indirect	Indirect	Indirect	***(Direct)***	Indirect	Indirect	Indirect	Indirect
False-Negative (2)						**(Indirect)**		***Direct***		**(Indirect)**		
**Potential Art Completion (Effect)***												
Direct Selection								Positive				
Indirect Selection						Positive				Positive		

*After artwork approval by the team, a positive effect is determined when the direct or indirect selected team wins the Super Bowl leading to the completion of the artwork.

Method of Everything Inequality (≤ 1) Data* © (2000-2012)

Experiment	#1	#2	#3	#4	#5	#6	#7	#8	#9	#10	#11	#12
NFL Season	2000/01	2001/02	2002/03	2003/04	2004/05	2005/06	2006/07	2007/08	2008/09	2009/10	2010/11	2011/12
NFL Teams	Totals	Totals	Totals	Totals	Totals	**Totals**	Totals	**Totals**	Totals	**Totals**	Totals	Totals
49ERS	286	66	80,703	32,959	54,050	1,531	1,305	6,562	12,812	10,437	3,668	***54,938***
BEARS	840	354	87,237	25,817	63,090	2,627	2,517	958	72,009	13,456	***169,000***	2,198
BENGALS	370	105	47,460	24,982	10,107	1,213	1,470	993	12,551	21,237	3,991	2,363
BILLS	36,169	56,372	***211,756***	***340,536***	191,264	29,789	31,892	12,067	90,822	33,862	17,689	15,481
BRONCOS	28,237	4,190	64,886	57,953	45,205	7,120	7,468	621	59,129	19,966	5,010	6,938
BROWNS	427	100	39,189	6,928	19,440	4,912	5,172	989	30,796	6,658	4,147	726
BUCCANEERS	4,243	1,078	71,816	20,949	8,988	7,561	7,757	247	42,915	6,570	4,840	900
CHARGERS	508	461	61,121	6,478	134,173	3,001	3,195	15,627	60,684	22,544	16,909	1,089
CARDINALS	315	185	81,386	7,016	8,498	5,551	5,930	792	238,436	22,884	4,445	559
CHIEFS	621	109	44,100	139,585	16,878	2,444	2,392	285	11,880	8,016	24,311	703
COLTS	4,226	989	57,475	91,467	127,812	1,452	1,358	8,928	79,441	75,675	23,212	639
COWBOYS	576	150	55,401	115,074	13,990	8,151	8,538	40,814	29,027	30,018	3,792	1,260
DOLPHINS	20,192	***136,211***	112,891	114,835	26,392	1,954	2,089	564	140,203	17,513	4,692	492
EAGLES	23,774	10,564	112,938	176,503	164,964	3,101	2,457	10,963	***544,615***	36,168	22,531	2,035
GIANTS	22,102	24,692	140,351	67,028	160,257	11,234	10,232	***176,415***	170,374	17,030	15,167	39,888
FALCONS	308	178	55,241	7,359	91,262	7,812	9,435	211	58,985	10,175	58,766	2,335
JAGUARS	2,025	125	42,994	6,188	31,262	3,618	4,430	35,894	35,894	7,796	5,300	586
JETS	4,505	23,060	108,250	69,228	251,871	3,451	3,620	1,147	98,794	86,322	112,315	1,203
LIONS	2,244	320	53,052	5,709	12,480	4,581	4,895	1,056	9,215	4,644	4,390	2,655
PACKERS	311	7,868	61,694	259,172	211,757	2,510	2,290	175,057	51,392	38,924	141,371	8,600
PANTHERS	325	227	50,082	95,427	22,693	2,310	2,160	896	102,619	6,680	4,009	771
PATRIOTS	845	151	78,302	72,559	53,135	2,101	1,937	15,360	37,325	11,438	84,404	24,686
RAIDERS	2,635	778	87,451	73,829	15,910	2,139	2,494	1,117	24,628	7,140	4,206	1,214
RAMS	***49,721***	111,093	191,151	277,767	238,297	38,989	40,993	27,098	88,987	11,648	80,073	597
RAVENS	2,609	1,918	48,584	25,046	23,278	3,101	3,207	1,102	203,483	24,900	46,812	54,885
REDSKINS	2,228	6,063	110,919	50,783	42,636	21,091	23,435	16,766	86,508	19,185	3,808	1,105
SAINTS	3,142	383	53,072	35,849	25,521	2,319	2,709	1,013	16,075	**80,921**	72,092	6,531
SEAHAWKS	486	100	68,112	62,438	53,196	20,211	21,286	15,075	20,789	6,548	37,865	762
STEELERS	1,203	448	67,068	20,789	227,894	**21,783**	23,731	4,502	498,026	12,766	143,290	2,133
TEXANS			37,238	8,052	12,798	6,799	7,695	1,034	24,554	10,334	6,248	5,121
TITANS	14,466	1,685	49,332	79,436	26,299	2,718	2,996	1,747	114,405	9,217	4,284	1,000
VIKINGS	4,084	1,127	58,724	220,192	***286,059***	***69,931***	***75,262***	172,453	181,974	***88,533***	9,980	466
**Super Bowl**	**XXXV**	**XXXVI**	**XXXVII**	**XXXVIII**	**XXXIX**	**XL**	**XLI**	**XLII**	**XLIII**	**XLIV**	**XLV**	**XLVI**
**TOTAL VOTES**	234,023	391,150	2,489,976	2,597,933	2,671,456	307,105	326,347	748,353	3,249,347	779,205	1,142,617	244,859
**15,182,371**												
**Selection Events (Cause)**								***Direct***		Indirect	Indirect	Indirect
False-Positive (1)	Indirect	Indirect	Indirect	Indirect	Indirect	Indirect	Indirect	*(Direct)*	Indirect	Indirect	Indirect	Indirect
False-Negative (2)						**(Indirect)**		***Direct***		**(Indirect)**		
Method of Everything ≤ 1 Inequality (Cause = Effect)*						**0.5**		**1**		**0.5**		
**Direct Selection (1-DS) ≤ 1 calculation of 12/1 = 12 = 1 (single-slit)**								**Positive**				
**Indirect Selection (2-IS) ≤ 1 calculation of 12/2 = 6 = .5 (double-slit)**						**Positive**				**Positive**		

*After artwork approval by the team, a positive effect is determined when the direct or indirect selected team wins the Super Bowl leading to the completion of the artwork.
