# Supplementary material for: The Method of Everything vs. Experimenter Bias of Loophole-Free Bell Experiments
Source: Front Res Metr Anal. 2024 Jul 11;9:1404371. doi: 10.3389/frma.2024.1404371 (PMC11269139; doi:10.3389/frma.2024.1404371)
Supplement: Data Sheet 3 — Gravity – the Fundamental Force of Nature. [file Data_Sheet_3.PDF]

Gravity - the Fundamental Force of Nature - $E = G^2$
(Method of Everything Supplement)

Manuel S Morales © 2023

Although the Cartesian coordinate system helped inspire my analysis of the unambiguous empirical evidence obtained from the Tempt Destiny Experiment, [1] the variables involved are not locally quantitative. Instead, the variables of motion are qualitative as part of a nonlocal dichotomy coordinated by their corresponding axis. The axiom analysis of direct selection and indirect selection functions [6] reveals their proper order and how gravity and energy can be understood as one.

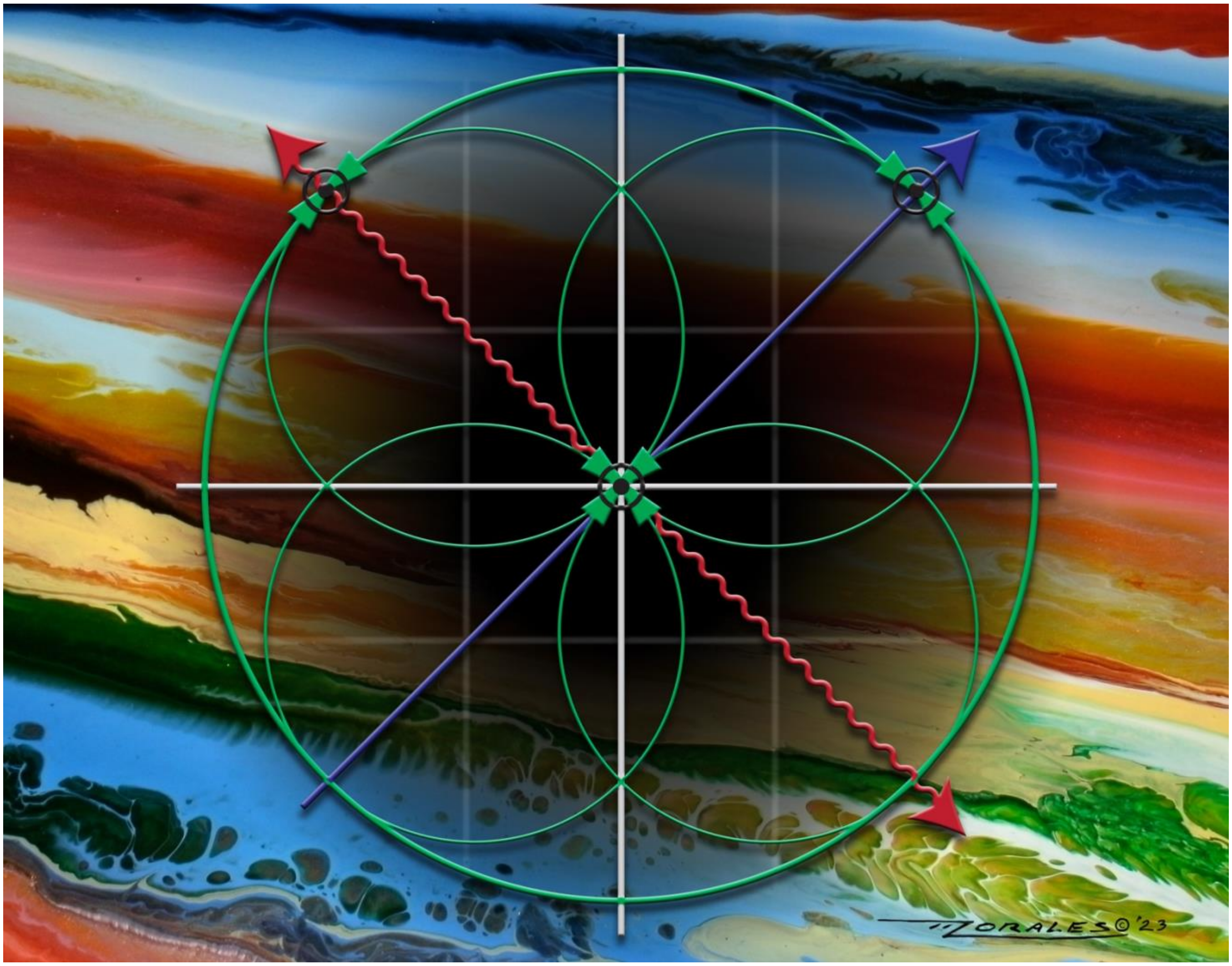

Gravity – Hidden Variables of Attraction:
As empirically confirmed, the hidden variables of motion (indicated by the above faint axioms), direct selection and indirect selection, can only *come-to-exist* (Table 3). As such, they are necessary nonlocal attraction events (center arrows) of the first order. As exemplified in the graph, a direct selection consists of strong motion (x) paired with a single potential (y) (center arrows, bottom left axiom), and indirect selection consists of weak motion (-x) paired with more-than-one potential (y) (center arrows, bottom right axiom). In other words, direct selection - motion is primary (strong), and indirect selection - motion is divided and thus not primary (weak).

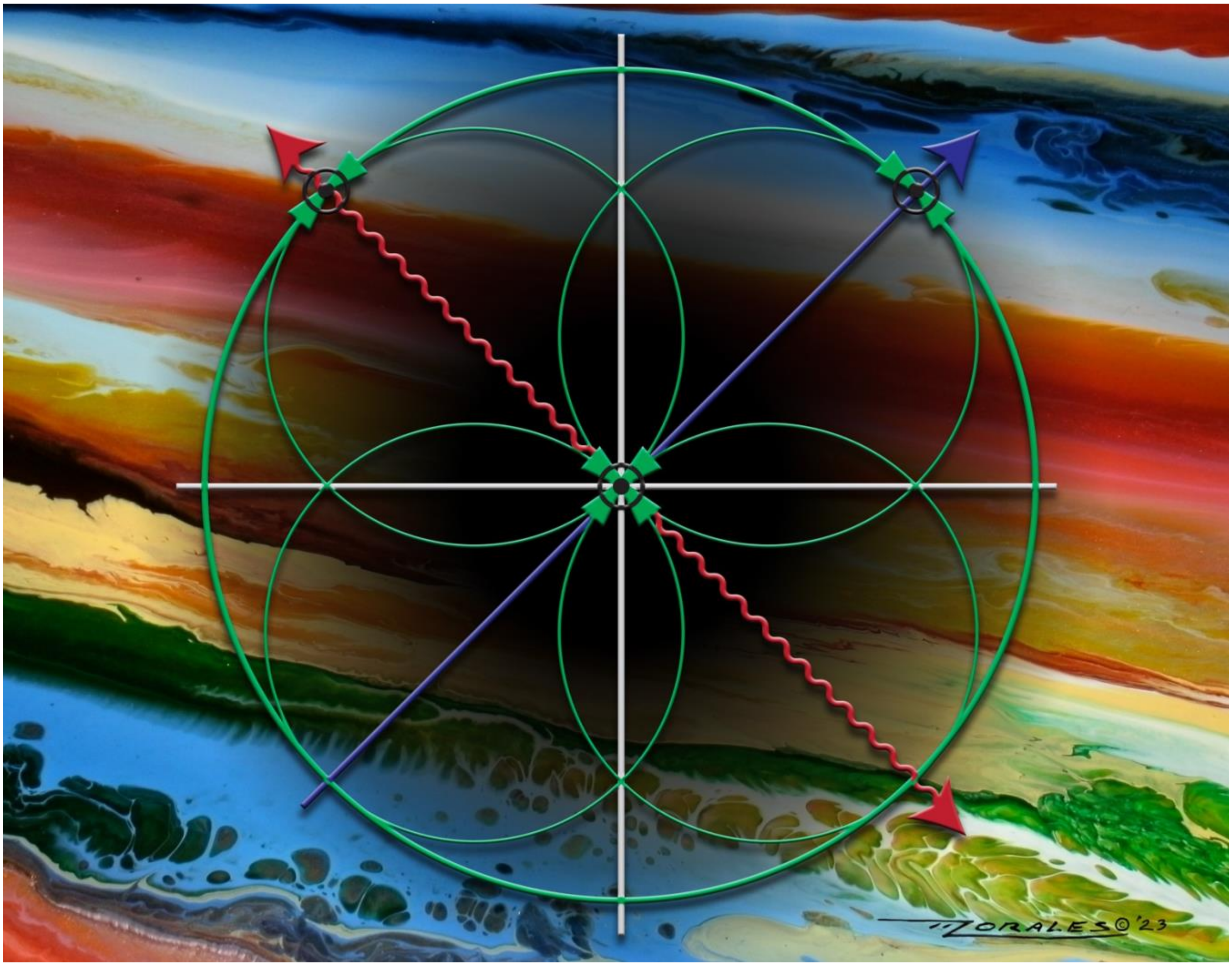

Momentum – Hidden Variables of Attraction:

- The effect of a nonlocal direct selection (z) can be locally understood as linear momentum (top right axiom (z) with the bottom left axiom (x, y) - blue arrow)
- The effect of a nonlocal indirect selection (-z) can be locally understood as angular momentum (top left axiom (-z) with bottom right axiom (-x, y) – red arrow).

As empirically confirmed without exception in the Tempt Destiny Experiment, a selection can only come-to-exist. As such, the attraction event of motion paired with a potential selection function is fundamental. This means that the attraction function known as gravity is primary (center arrows) and its effects (external arrows), i.e., strong electromagnetic force (blue arrow) and weak electromagnetic force (red arrows), are secondary effects – momentum. [see reference 6, pp. 6-9]

The four underlying axiom coordinate systems combine to create one axiom coordinate system, i.e., an object of motion, is held together by the fundamental attraction of both direct selection (+g) and indirect selection (-g). Hence – $\boldsymbol{E = G^2}$

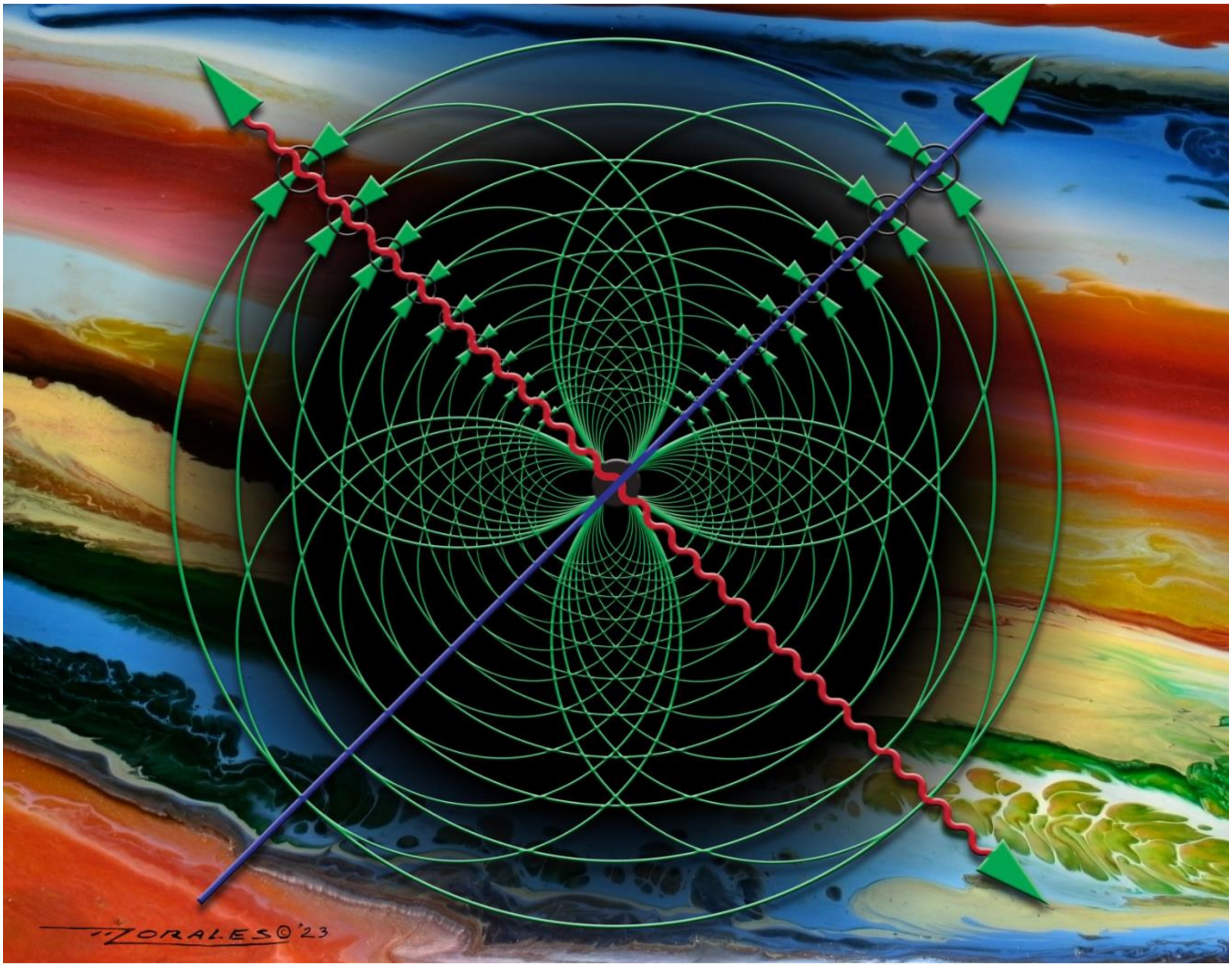

Evolution of Motion:
As the Final Selection Experiment has confirmed, states of physical existence are objects of motion. Understand this and we can then understand how physical existence evolves from one state of existence to another via Choice/Chance Mechanics. [1]

This is how nature gets something from nothing.
