# Supplementary figures and images for: The Method of Everything vs. Experimenter Bias of Loophole-Free Bell Experiments
Source: Front Res Metr Anal. 2024 Jul 11;9:1404371. doi: 10.3389/frma.2024.1404371 (PMC11269139; doi:10.3389/frma.2024.1404371)

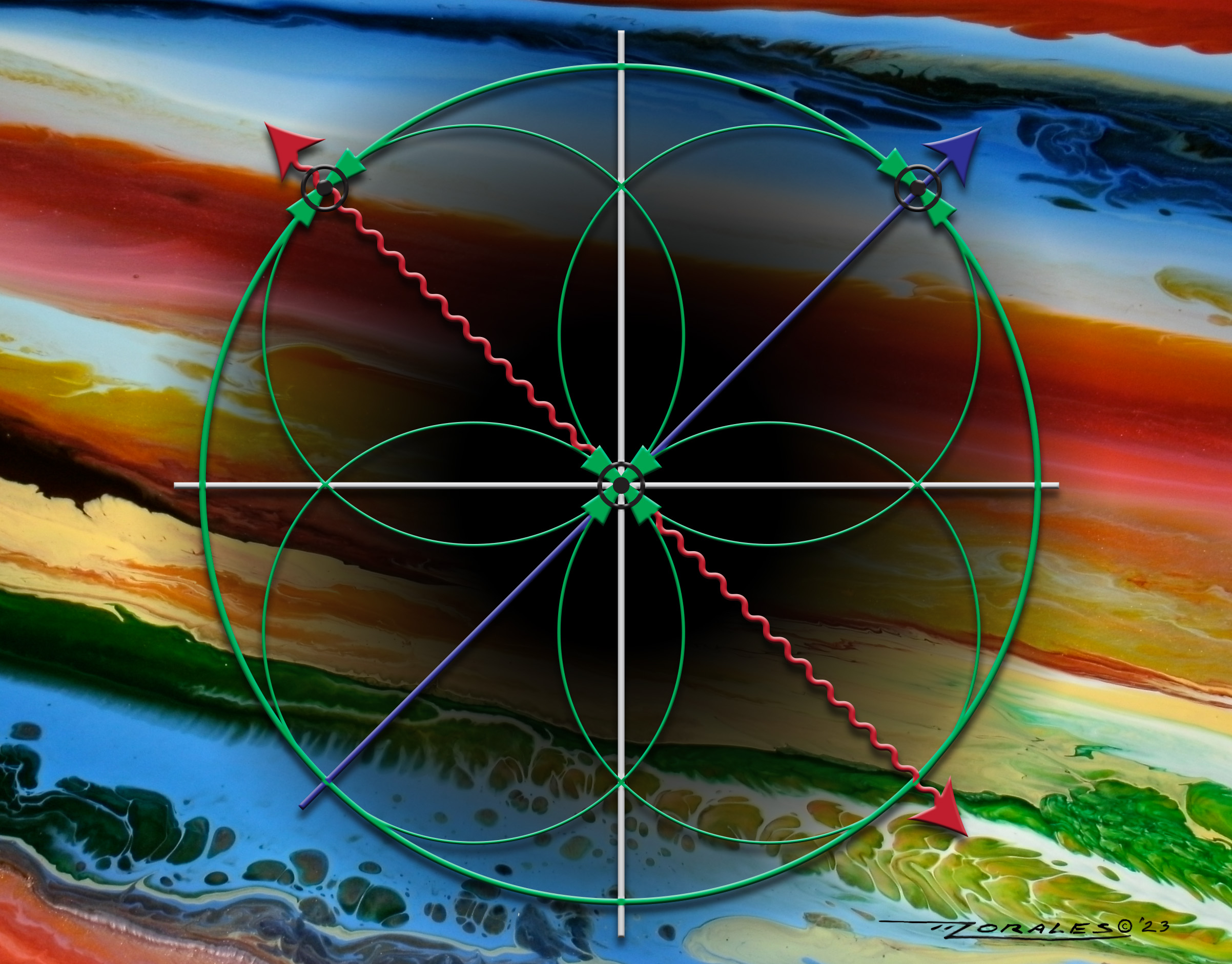

Supplement: Image 1 — E = G2. [file Image_1.JPEG]
